# Author Correction: A framework for clinical cancer subtyping from nucleosome profiling of cell-free DNA

**DOI:** 10.1038/s41467-023-36187-8

**Published:** 2023-01-25

**Authors:** Anna-Lisa Doebley, Minjeong Ko, Hanna Liao, A. Eden Cruikshank, Katheryn Santos, Caroline Kikawa, Joseph B. Hiatt, Robert D. Patton, Navonil De Sarkar, Katharine A. Collier, Anna C. H. Hoge, Katharine Chen, Anat Zimmer, Zachary T. Weber, Mohamed Adil, Jonathan B. Reichel, Paz Polak, Viktor A. Adalsteinsson, Peter S. Nelson, David MacPherson, Heather A. Parsons, Daniel G. Stover, Gavin Ha

**Affiliations:** 1 Division of Public Health Sciences and Human Biology, Fred Hutchinson Cancer Center, Seattle, WA USA; 2 Molecular and Cellular Biology Graduate Program, University of Washington, Seattle, WA USA; 3 Medical Scientist Training Program, University of Washington, Seattle, WA USA; 4 Department of Genome Sciences, University of Washington, Seattle, WA USA; 5 Dana-Farber Cancer Institute, Boston, MA USA; 6 Division of Medical Oncology, Department of Medicine, University of Washington, Seattle, WA USA; 7 Ohio State University Comprehensive Cancer Center, Columbus, OH USA; 8 Laboratory Medicine and Pathology, University of Washington, Seattle, WA USA; 9 Brotman Baty Institute for Precision Medicine, Seattle, WA USA; 10 Department of Oncological Sciences, Icahn School of Medicine, Mount Sinai, New York, NY USA; 11 Broad Institute of MIT and Harvard, Cambridge, MA USA

**Keywords:** Breast cancer, Tumour heterogeneity, Machine learning, Diagnostic markers, Cancer genomics

Correction to: *Nature Communications* 10.1038/s41467-022-35076-w, published online 03 December 2022

In this article the patent application mentioned in the ‘Competing interests’ statement was incorrectly given as ‘PCT/US2002/024082’ but should have been ‘PCT/US2022/024082’. The original article has been corrected.

