# Supplementary material for: A framework for clinical cancer subtyping from nucleosome profiling of cell-free DNA
Source: Nat Commun. 2022 Dec 3;13:7475. doi: 10.1038/s41467-022-35076-w (PMC9719521; doi:10.1038/s41467-022-35076-w)
Supplement: Supplementary file 1 — Supplementary Figures [file 41467_2022_35076_MOESM1_ESM.pdf]

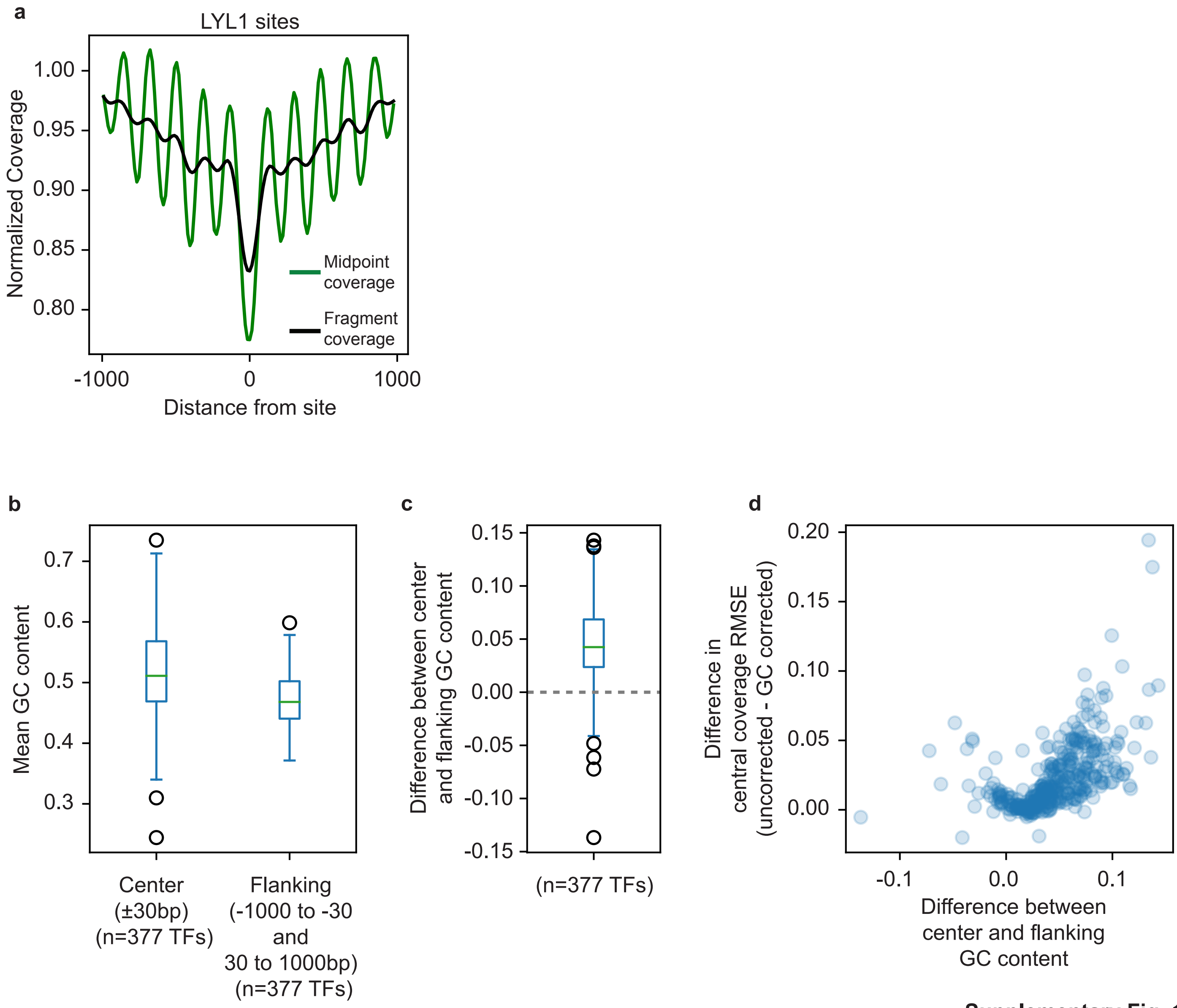

Supplementary Fig. 1

Supplementary Fig. 1: (a) Comparison of midpoint coverage profiles with fragment coverage profiles. The midpoint coverage profile was computed using Griffin for the top 10,000 LYL1 sites in a healthy donor sample (HD_45). Griffin computes coverage by counting the number of midpoints that overlap each site. The fragment coverage was computed using a modified version of Griffin which counted the number of fragments overlapping each position. (**b)** Boxplot of the mean GC content around the top 10,000 TFBSs for each of 377 TFs. For each TFBS, the GC content was calculated in two windows: center (±30bp from the TFBS) and flanking (±1000bp from the TFBS, excluding the center region ±30bp). The boxed range represents the median ± IQR of the 377 TFs, whiskers represent the range of the non-outlier data (maximum extent is 1.5x the IQR). Outliers are plotted as circles. (**c)** Boxplot of the difference between the mean center GC content and mean flanking GC content for each of the 377 TFs. Box elements are the same as in (b). (**d)** Scatter plot of the difference between center and flanking GC content and the difference in the RMSE before and after GC correction. Source data are provided as a Source Data file.

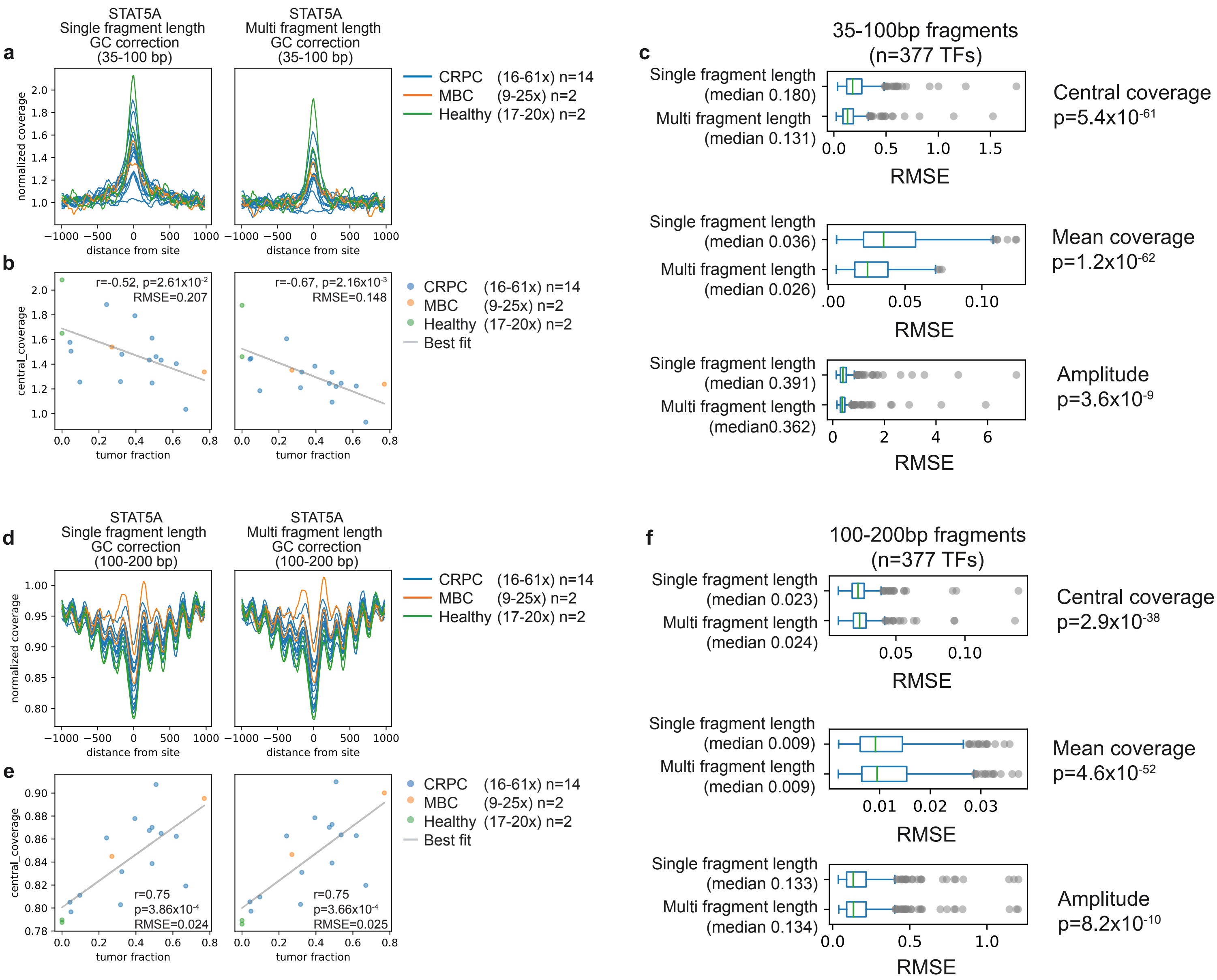

Supplementary Fig. 2

Supplementary Fig. 2: (a) Composite coverage profiles derived from short cfDNA fragments (35-100bp) of 10,000 STAT5A sites with single fragment length (left) and multi fragment length (right) GC correction approaches (see methods), shown for 14 CRPC samples with deep WGS (16-61x WGS, blue), two MBC samples (9-25x WGS, orange), and two healthy donors (17-20x WGS, green). Short cfDNA fragments are derived from TF sites that are actively bound and protected by the TF itself[1]. For STAT5A, which is associated with hematopoiesis[2], we observe the expected increase in coverage at binding sites in healthy donors and lower tumor fraction samples. **(b)** cfDNA tumor fraction and short fragment central coverage correlation for STAT5A, shown for the same deep WGS samples as in (a). cfDNA contains a mixture of tumor and blood cells; therefore, central coverage values are expected to be negatively correlated with tumor fraction for STAT5A (higher coverage represents increased TF binding). The multi fragment length approach leads to a stronger correlation based on Pearson's r correlation coefficient and p-value (2 sided). Root mean squared error (RMSE) of the linear fit is shown. **(c)** Boxplots showing the distribution of the RMSE (linear fit between each of the three features for short fragments (35-100bp) and tumor fraction across the 377 TFs, for single fragment length and multi fragment length GC correction for the same deep WGS samples as in (a). For central coverage 360 of 377 TFs had lower RMSE with multi fragment length GC correction (95%), for mean coverage 366 of 377 (97%) and for amplitude 226 of 377 (60%). p-values were calculated using the Wilcoxon signed-rank test (two-sided). The boxed range represents the median ± IQR, whiskers represent the range of the non-outlier data (maximum extent is 1.5x the IQR). Outliers are plotted in grey. **(d)** Composite coverage profiles derived from nucleosome sized cfDNA fragments (100-200bp) of 10,000 STAT5A sites with single fragment length (left) and multi fragment length (right) GC correction approaches, shown for the same samples as (a). For nucleosome sized fragments, lower 'central coverage' corresponding to greater site accessibility in the healthy donor samples is expected because STAT5A is a transcription factor associated with hematopoiesis. **(e)** cfDNA tumor fraction and short fragment central coverage correlation for STAT5A, shown for the same samples as in (a). Central coverage values for nucleosome sized fragments are expected to be positively correlated with tumor fraction for STAT5A (lower represents greater accessibility). Root mean squared error (RMSE) of the linear fit is shown. **(f)** Boxplots showing the distribution of the RMSE (linear fit between each of the three features for nucleosome sized fragments (100-200bp) and tumor fraction across the 377 TFs, for single fragment length and multi fragment length GC correction. For central coverage 86 out of 377 TFs had lower RMSE after correction (95%), for mean coverage 44 of 377 (97%) and for amplitude 145 of 377 (60%). p-values were calculated using the Wilcoxon signed-rank test (two-sided). Box elements are the same as in (c). Source data are provided as a Source Data file.

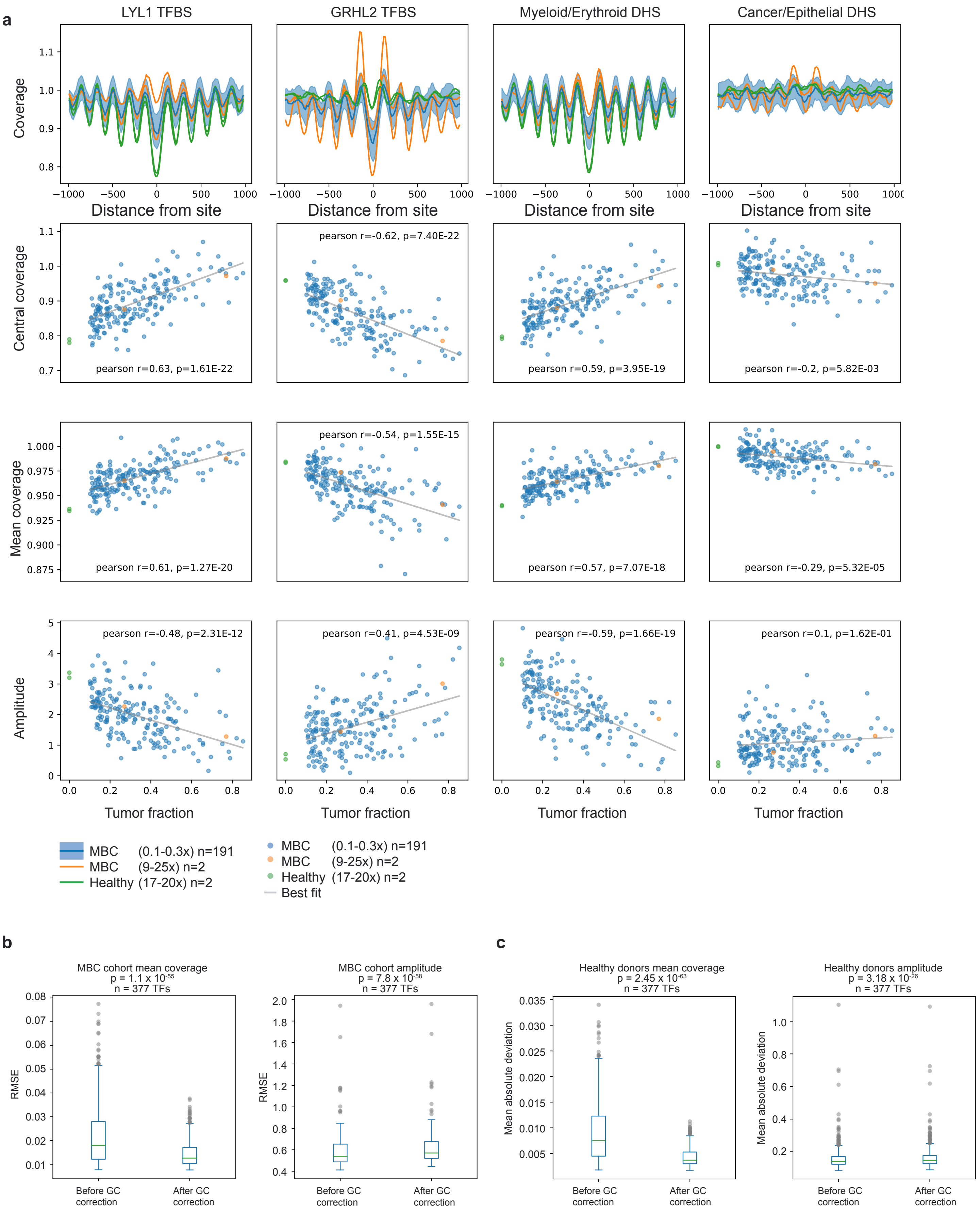

Supplementary Fig. 3

Supplementary Fig. 3: Correlations between tumor fraction and features. **(a)** Top row: Nucleosome profiles for 4 site types that are healthy blood specific (LYL1 and Myeloid/Erythroid DNase Hypersensitivity Sites (DHS)) or cancer specific (GRHL2 and Cancer/Epithelial DHS). Each profile is from the top 10,000 sites. For 191 ULP-WGS MBC samples with ≥0.1 tumor fraction,[3] the median coverage ± IQR is shown. Two healthy donor samples and two deep-WGS MBC samples are included for illustration. Second, third, and fourth rows: Correlation between tumor fraction and central coverage (second row), mean coverage (third row), and amplitude (fourth row) for the 191 MBC samples. Pearson's r and p-value (two sided) are shown for each correlation. Because central coverage and mean coverage are reduced when a site is accessible, these features are positively correlated with tumor fraction for blood specific sites and negatively correlated with tumor fraction for cancer specific sites. Amplitude is increased when a site is accessible, so this feature is expected to be negatively correlated with tumor fraction for blood specific sites and positively correlated with tumor fraction for tumor specific sites. For all plots, healthy and deep-WGS MBC samples are included for illustration and not included in statistics. **(b)** Boxplots showing the distribution of the RMSE (linear fit between mean coverage and tumor fraction in the MBC ULP-WGS dataset [0.1-0.3x, n=191]) across the 377 TFs, before and after GC correction. The boxed range represents the median ± IQR, whiskers represent the range of the non-outlier data (maximum extent is 1.5x the IQR). Outliers are plotted in grey. p-value was calculated using the Wilcoxon signed-rank test (two-sided). 325 of 377 TFs (86%) have a lower RMSE post GC correction for mean coverage and 32 of 377 (8.5%) for amplitude. **(c)** Boxplots showing the distribution of the mean absolute deviation (of the mean coverage and amplitude across 215 healthy donors [1-2x WGS]) across the 377 TFs, before and after GC correction. Box elements are the same as (b). p-value was calculated using the Wilcoxon signed-rank test (two-sided). 372 of 377 TFs (99%) have a lower RMSE post GC correction for mean coverage and 89 of 377 (24%) for amplitude. Source data are provided as a Source Data file.

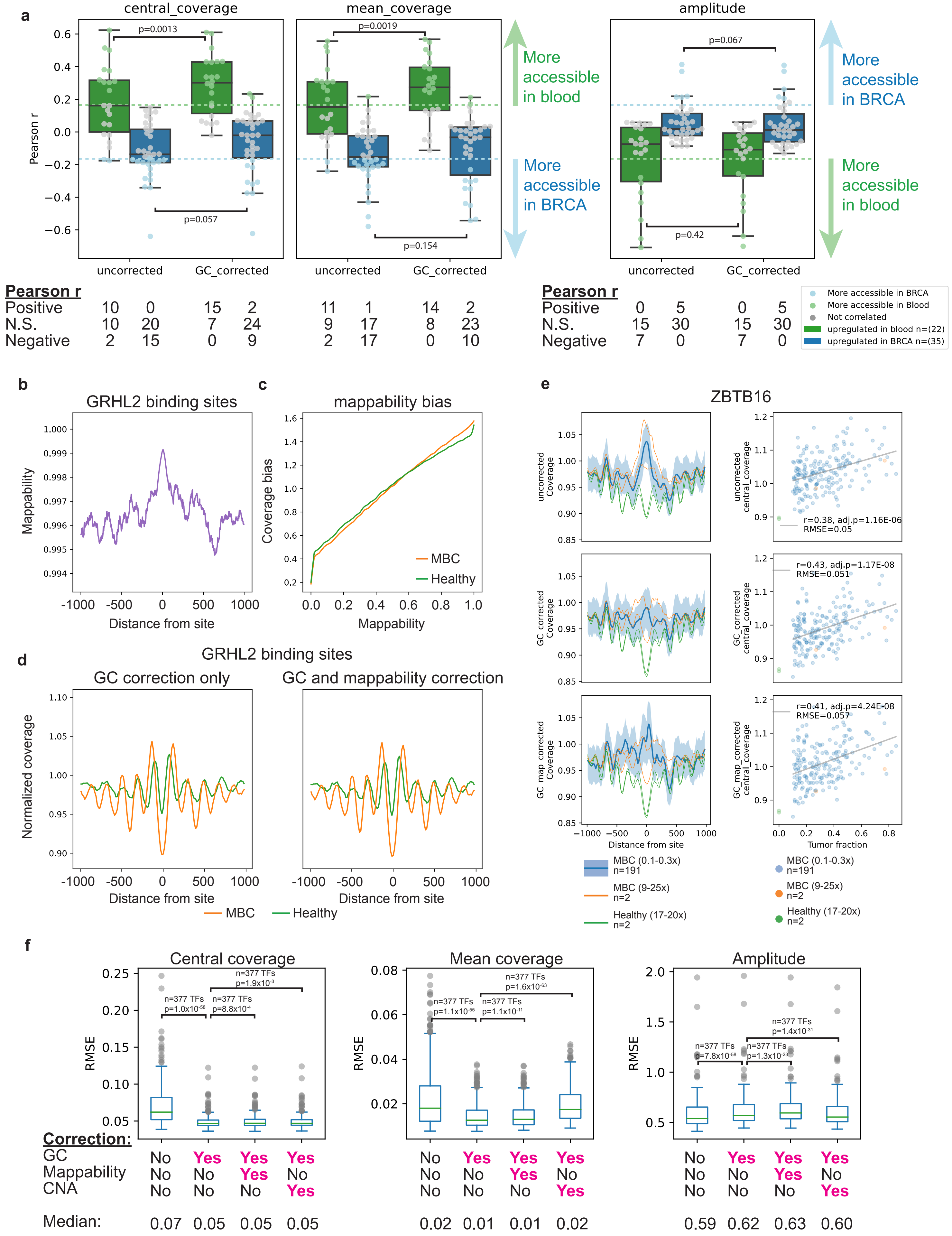

Supplementary Fig. 4

Supplementary Fig. 4: (a) Boxplots of Pearson correlation coefficients for TFs that are differentially expressed between blood cells and breast cancer (BRCA) cells. TFs were identified using differential gene expression analysis and further filtered to remove TFs which shared many sites with differential TFs from the opposite group (methods). After these filters, there were a total of n=22 blood TFs (upregulated in blood relative to cancer, green boxes) and n=35 BRCA TFs (upregulated in cancer relative to blood, blue boxes). Individual values for correlation coefficients between tumor fraction and cfDNA features (centeral coverage, mean coverage, or amplitude) for n=191 metastatic breast cancer samples were plotted. A subset of TFs had cfDNA features that were significantly correlated with tumor faction and these correlations tended to be in the expected direction. Points were colored green (more accessible in blood) or blue (more accessible in cancer) if the Pearson correlation was significant ($p < 0.05$, two-sided) after FDR correction. For TFs that were upregulated in blood, central coverage, and mean coverage (left and middle panels) tended to be positively correlated with tumor fraction indicating more accessibility in blood cells and GC correction significantly increased this correlation ($p = 0.0013$ for central coverage and $p = 0.0019$ for mean coverage, Wilcoxon signed rank test, two sided). Amplitude was negatively correlated with tumor fraction, as expected, but not significantly impacted by GC correction ($p = 0.42$, Wilcoxon signed rank test, two sided). For TFs that were upregulated in BRCA, the opposite trend was observed with negative correlations for mean coverage and central coverage and a positive correlation for amplitude. GC correction did not significantly impact these correlations. Box elements are the same as (f). **(b)** Aggregated mean mappability at 10,000 GRHL2 binding sites and its surrounding 2kb region showing a slight increase in mappability (Umap multi-read mappability track for 100bp) at the site center. **(c)** cfDNA mappability bias is unique to each sample. Mappability bias computed for cfDNA from a healthy donor (HD_46; green) and a metastatic breast cancer (MBC_315; orange). **(d)** Composite coverage profile of 10,000 GRHL2 binding sites before and after mappability correction, shown for HD_46 (green) and MBC_315 (orange). There is minimal change in coverage profile after mappability correction. **(e)** Composite coverage profiles of 10,000 ZBTB16 sites before correction (top row), after GC correction only (middle row) and after GC and mappability correction (bottom row), shown for two MBC samples with deep WGS (9-25x, orange), two healthy donors (17-20x, green), and 191 MBC samples with ULP-WGS (0.1-0.3x, blue). Median +/- IQR of 191 ULP-WGS samples is shown with blue shading. Lower 'central coverage' corresponding to greater site accessibility in the healthy donor samples is expected because ZBTB16 is a transcription factor associated with hematopoiesis[4]. However, the addition of mappability correction leads to increased noise (unexpected spikes in coverage) relative to the GC corrected profile. After GC correction, the correlation between tumor fraction and central coverage (for the MBC ULP-WGS samples) is stronger based on Pearson's r correlation coefficient (two sided), however, the addition of mappability correction (bottom row) weakens the correlation. **(f)** Boxplots showing the distribution of the RMSE (Root mean squared error; linear fit between central coverage and tumor fraction in the MBC ULP-WGS dataset [0.1-0.3x, n=191]) across the n=377 TFs, before and after GC, mappability, and copy number alteration (CNA) correction. GC correction leads to a large improvement (decrease) in median RMSE for central coverage and mean coverage but not amplitude (Figure 2f, Supplementary Fig. 3b). However, mappability and CNA correction both have a more modest effect on all features leading to a slight but significant increase in RMSE for all features except amplitude at CNA corrected sites. The boxed range represents the median ± IQR, whiskers represent the range of the non-outlier data (maximum extent is 1.5x the IQR). Outliers are plotted in grey. p-values were calculated using the Wilcoxon signed-rank test (two-sided). Source data are provided as a Source Data file.

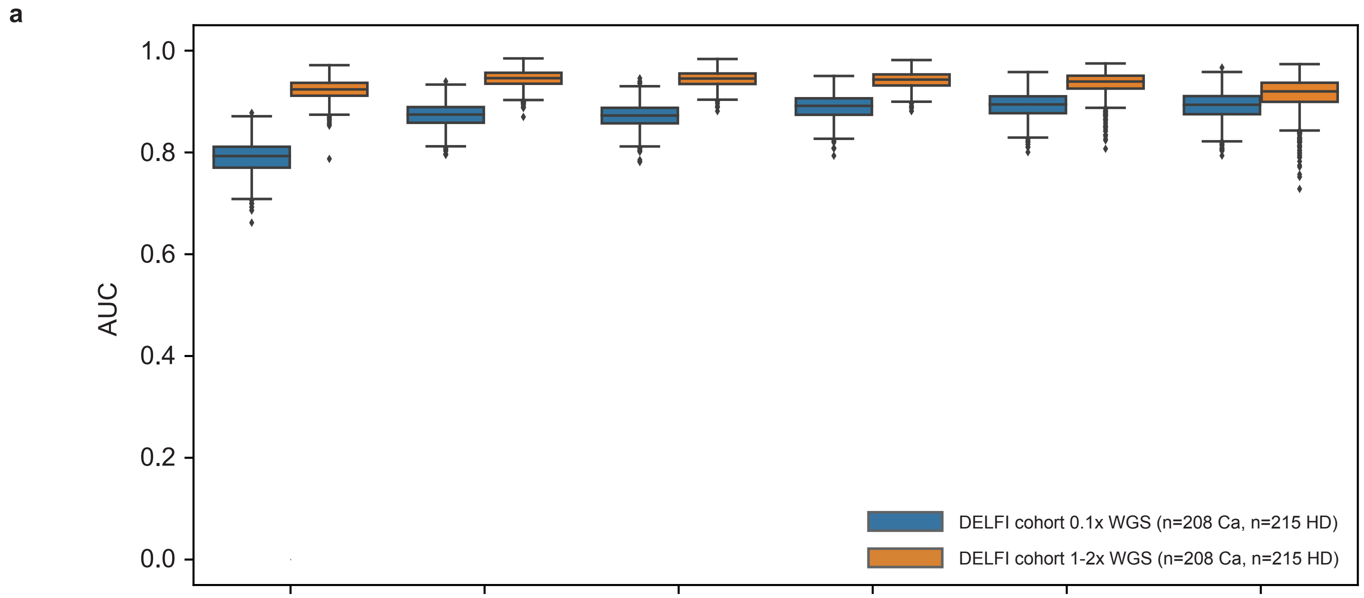

		1000-sites	5000-sites	10000-sites	20000-sites	30000-sites	50000-sites
**Number of sites**		1000-sites	5000-sites	10000-sites	20000-sites	30000-sites	50000-sites
**Number of TFs**		566	446	377	316	270	202
**AUC (95% CI)**	**0.1x WGS**	0.793 (0.726-0.847)	0.874 (0.819-0.917)	0.873 (0.823-0.915)	0.892 (0.840-0.933)	0.894 (0.839-0.936)	0.894 (0.827-0.938)
	**1-2x WGS**	0.924 (0.884-0.958)	0.946 (0.903-0.974)	0.945 (0.910-0.970)	0.943 (0.907-0.969)	0.939 (0.876-0.966)	0.920 (0.820-0.958)

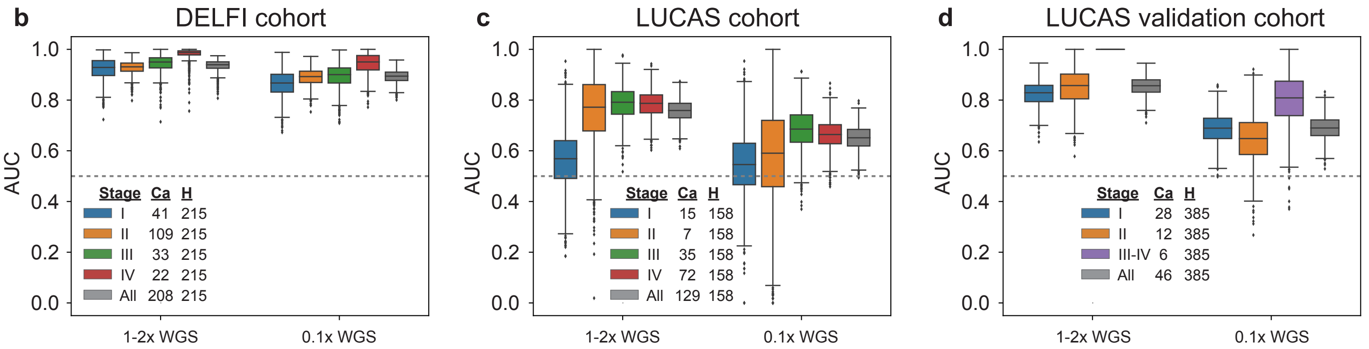

Supplementary Fig. 5

Supplementary Fig. 5: Cancer detection performance metrics. **(a)** Distribution of AUC values for 1,000 bootstrap iterations of the cancer detection logistic regression model on the DELFI cohort (n=208 cancer (Ca) cfDNA samples, n=215 healthy donor (H) cfDNA samples) using different numbers of TFBSs per TF when running Griffin (methods). Performance for 1-2x WGS data (orange) and 0.1x downsampled WGS data (blue) are shown. The boxed range represents the median ± IQR, whiskers represent the range of the non-outlier data (maximum extent is 1.5x the IQR). Outliers are plotted as grey diamonds. 95% confidence intervals (CIs) are printed below the plot and were obtained from 1,000 bootstrap iterations **(b)** Distribution of AUC values for 1,000 bootstrap iterations of the cancer detection logistic regression model on the DELFI cohort [same samples as in (a)] for 1-2x WGS data and 0.1x WGS data. Values are shown for each stage and overall. Corresponds to the data and values shown in Main Figure 3a. Box elements are the same as in (a). **(c)** Distribution of AUC values for 1,000 bootstrap iterations of the cancer detection logistic regression model on the LUCAS cohort (n=129 cancer (Ca) cfDNA samples, n=158 non-cancer (H) cfDNA samples) for 1-2x WGS data and 0.1x WGS data. Values are shown for each stage and overall. Corresponds to the data and values shown in Main Figure 3c. Box elements are the same as in (a). **(d)** Distribution of AUC values for 1,000 bootstrap iterations of the cancer detection logistic regression model on the LUCAS validation cohort (n=28 cancer (Ca) cfDNA samples, n=385 healthy donor (H) cfDNA samples) for 1-2x WGS data and 0.1x WGS data. Values are shown for each stage (III and IV are combined due to small number of samples) and overall. Corresponds to the data and values shown in Main Figure 3d. Box elements are the same as in (a). Source data are provided as a Source Data file.

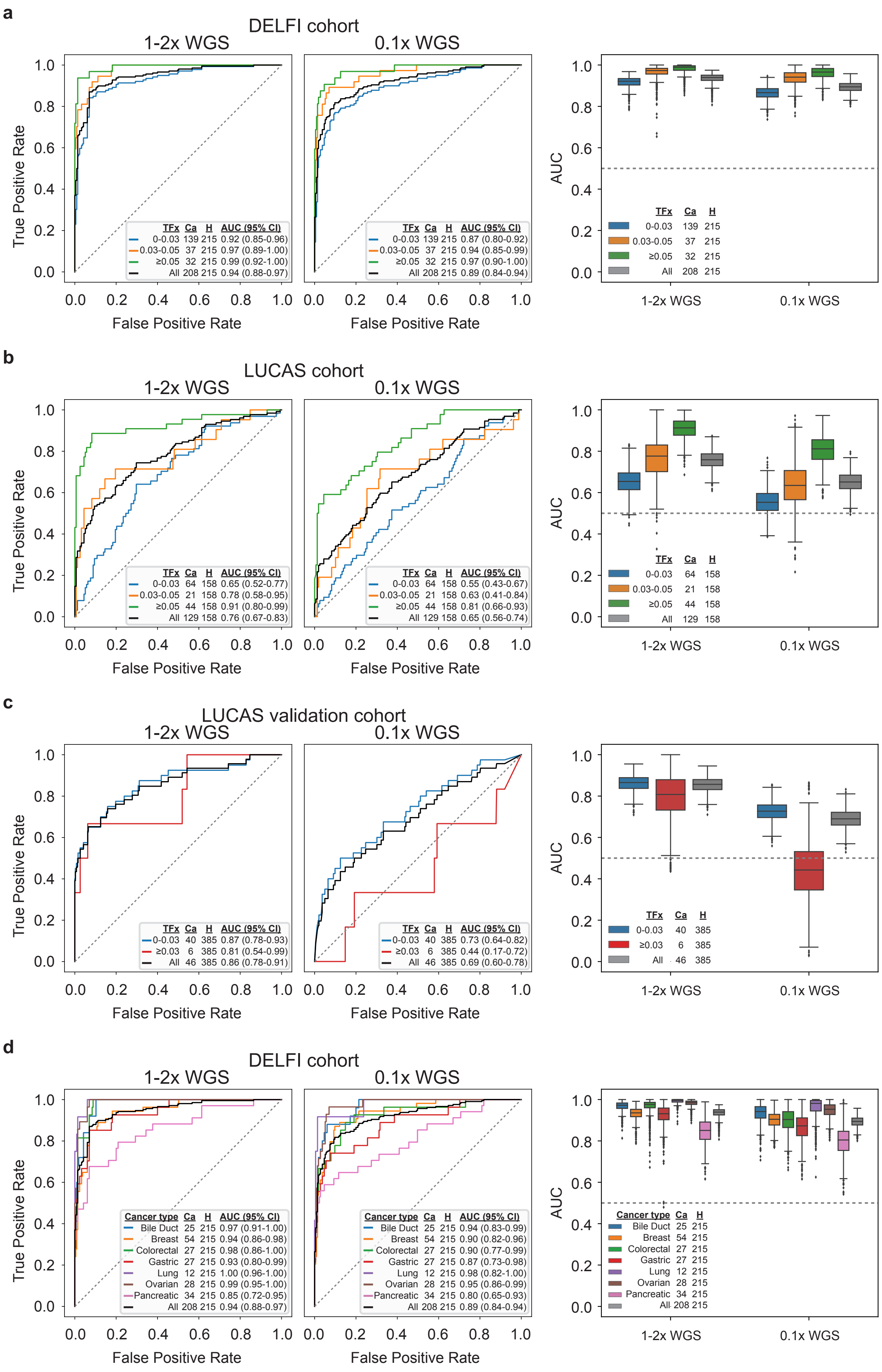

Supplementary Fig. 6

Supplementary Fig. 6: Cancer detection performance metrics by tumor fraction (TFx) and cancer type. Receiver operator characteristic (ROC) curves for logistic regression classification of cancer (Ca) vs. healthy controls (H) are shown for three cohorts, **(a,d)** the DELFI cohort[5], **(b)** the LUCAS cohort, and **(c)** the LUCAS validation cohort[6]. Logistic regression was performed on the top PCA components which explained 80% of the variance in the features (central coverage, mean coverage, and amplitude) extracted from nucleosome profiles around 30,000 TFBSs for each of 270 TFs. ROC for cancer grouped by TFx vs. healthy **(a-c)** or cancer grouped by cancer type vs. healthy **(d)** are shown. Duodenal cancer (n=1) is not shown as a separate cancer type. For each cohort, performance is shown for both the original low pass (1-2x) WGS (left panel) and ultra-low pass (0.1x) WGS (middle panel) generated by in-silico downsampling. 95% confidence intervals (CIs) were obtained from 1,000 bootstrap iterations. The right panel for each cohort contains boxplots of the AUC values for the bootstrap iterations. The boxed range represents the median ± IQR, whiskers represent the range of the non-outlier data (maximum extent is 1.5x the IQR). Outliers are shown as grey diamonds. Source data are provided as a Source Data file.

a

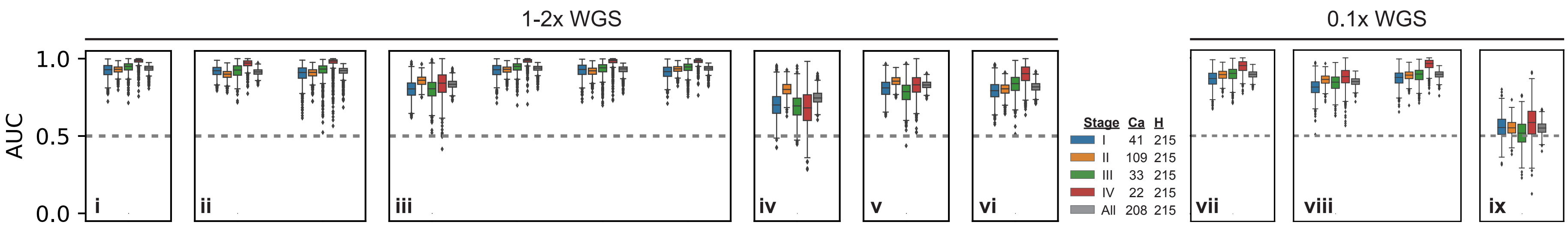

	i	ii	ii	iii	iii	iii	iii	iv	v	vi	vii	viii	viii	ix
Fragment size	100-200bp	**35-150bp**	**35-500bp**	100-200bp	100-200bp	100-200bp	100-200bp	100-200bp	100-200bp	**All lengths**	100-200bp	100-200bp	100-200bp	**All lengths**
GC correction	Yes	Yes	Yes	**No**	Yes	Yes	Yes	Yes	Yes	**No**	Yes	**No**	Yes	**No**
Mappability correction	No	No	No	No	**Yes**	No	No	No	No	No	No	No	No	No
CNA correction	No	No	No	No	No	**Yes**	No	No	No	**Yes**	No	No	**Yes**	**Yes**
GC correction strategy	Multi-length	Multi-length	Multi-length	**N/A**	Multi-length	Multi-length	**Single-length**	Multi-length	Multi-length	**N/A**	Multi-length	**N/A**	Multi-length	**N/A**
Amplitude included	Yes	Yes	Yes	Yes	Yes	Yes	Yes	**No**	Yes	Yes	Yes	Yes	Yes	Yes
Site Type	TFBS	TFBS	TFBS	TFBS	TFBS	TFBS	TFBS	TFBS	**DHS**	**TFBS**	TFBS	TFBS	TFBS	**TFBS**
Tool used	Griffin	Griffin	Griffin	Griffin	Griffin	Griffin	Griffin	Griffin	Griffin	**Ulz et al.**	Griffin	Griffin	Griffin	**Ulz et al.**

AUC (95% Conf. Int.)

	finalized settings	short-fragment	all-fragments	no-GC-corr.	GC-map-corr.	CNA-corr.	single-len.-GC	no-amplitude	DHS	Ulz et al.
I	0.93 (0.83-0.98)	0.92 (0.84-0.98)	0.91 (0.77-0.98)	0.80 (0.69-0.91)	0.93 (0.83-0.99)	0.93 (0.83-0.98)	0.92 (0.82-0.98)	0.70 (0.54-0.86)	0.81 (0.69-0.91)	0.79 (0.67-0.90)
II	0.93 (0.87-0.97)	0.90 (0.83-0.95)	0.91 (0.82-0.96)	0.86 (0.79-0.92)	0.93 (0.87-0.97)	0.92 (0.84-0.96)	0.93 (0.88-0.97)	0.80 (0.72-0.89)	0.85 (0.79-0.91)	0.81 (0.71-0.87)
III	0.95 (0.86-0.99)	0.93 (0.83-0.99)	0.93 (0.79-0.98)	0.81 (0.65-0.93)	0.95 (0.86-0.99)	0.94 (0.83-0.99)	0.95 (0.86-0.99)	0.69 (0.53-0.85)	0.79 (0.63-0.90)	0.84 (0.70-0.94)
IV	0.99 (0.92-1.00)	0.97 (0.90-1.00)	0.98 (0.79-1.00)	0.84 (0.65-0.98)	0.99 (0.92-1.00)	0.99 (0.88-1.00)	0.99 (0.92-1.00)	0.68 (0.45-0.92)	0.83 (0.67-0.97)	0.90 (0.75-0.99)
All	0.94 (0.88-0.97)	0.91 (0.87-0.95)	0.92 (0.81-0.96)	0.83 (0.78-0.89)	0.94 (0.88-0.97)	0.93 (0.85-0.97)	0.94 (0.89-0.97)	0.75 (0.67-0.85)	0.83 (0.78-0.88)	0.82 (0.75-0.87)

	finalized settings	no-GC-corr.	CNA-corr.	Ulz et al.
I	0.87 (0.75-0.95)	0.81 (0.69-0.91)	0.87 (0.76-0.95)	0.55 (0.41-0.70)
II	0.89 (0.82-0.95)	0.86 (0.78-0.92)	0.89 (0.82-0.95)	0.55 (0.46-0.65)
III	0.90 (0.78-0.97)	0.84 (0.73-0.94)	0.90 (0.78-0.97)	0.52 (0.35-0.69)
IV	0.95 (0.84-1.00)	0.88 (0.73-0.99)	0.96 (0.87-1.00)	0.59 (0.36-0.79)
All	0.89 (0.84-0.94)	0.85 (0.79-0.90)	0.89 (0.84-0.94)	0.55 (0.47-0.63)

b

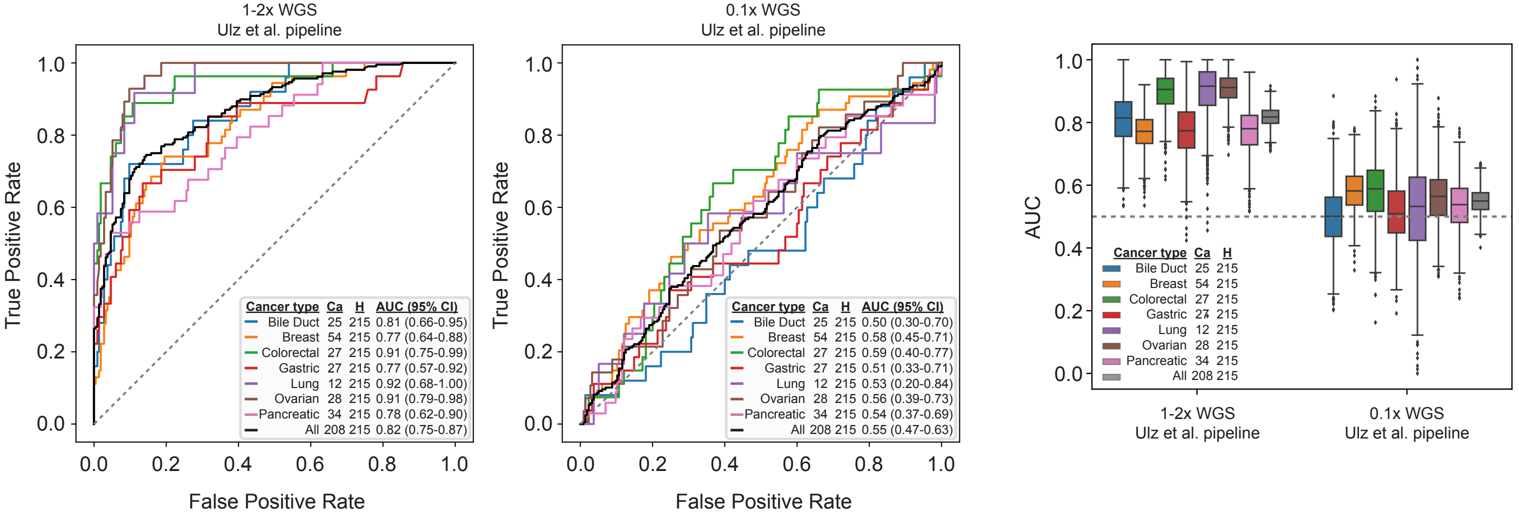

Supplementary Fig. 7

Supplementary Fig. 7: Evaluation of various configurations and comparisons of Griffin for cancer detection. **(a)** Boxplots of the AUC values for 1,000 bootstrap iterations of the logistic regression classifier using various configurations and comparisons of Griffin on the DELFI cohort (n=208 cancer (Ca) cfDNA samples, n=215 healthy donor (H) cfDNA samples) in the original 1-2x WGS data and downsampled data (0.1x WGS) grouped by stage. The boxed range represents the median ± IQR, whiskers represent the range of the non-outlier data (maximum extent is 1.5x the IQR). Outliers are shown as grey diamonds. **(i)** finalized Griffin configuration with parameters settings and median AUC for the 1,000 bootstraps (with 95% CI) listed underneath for comparison to other configurations. Logistic regression model was trained on top PCA features extracted from mean nucleosome profiles around 30,000 TFBSs for each of 270 TFs (total of 810 features prior to PCA dimensionality reduction), see methods. **(ii)** same Griffin analysis as in (i) but using two different fragment size ranges, short fragments, which are known to be enriched in cancer[7,8] (35-150bp), and a wider range of fragment sizes encompassing most cfDNA fragments (35-500bp). Both have decreased performance (0.91 and 0.92 AUC, respectively). **(iii)** Same Griffin analysis as in (i) but without GC correction (left), with an added mappability correction step (middle left), with an added CNA correction step (middle right), and with a different GC correction approach using a single fragment length (right). **(iv)** Same as (i) but with the amplitude features excluded from the model (only used central coverage and mean coverage features). **(v)** Griffin with standard parameters from (i) applied to the top 10,000 sites around 16 types of tissue specific DNAse hypersensitivity sites (see methods). **(vi)** Pipeline developed by Ulz and colleagues. This pipeline uses all fragment sizes (the vast majority of which are between 35 and 500bp) and collects coverage profiles around the top 1,000 sites for 504 transcription factors and extracts one feature (High frequency range) per feature. Dimensionality was reduced with PCA using the same approach as described in (i) and the top features were put into the logistic regression model. **(vii-xi)** Performance of selected configurations on 0.1x WGS data. **(b)** Receiver operator characteristic (ROC) curve for logistic regression classification of cancer vs. healthy controls using the pipeline from Ulz et al on the DELFI cohort[5] in 1-2x WGS data (left) and 0.1x WGS data (middle). ROC for each cancer type vs. healthy are shown. 95% confidence intervals (CIs) were obtained by bootstrapping. Duodenal cancer (n=1) is not shown as a separate cancer type. The right panel for each cohort contains boxplots of the AUC values for 1,000 bootstrap iterations. Box elements are the same as in (a). Source data are provided as a Source Data file.

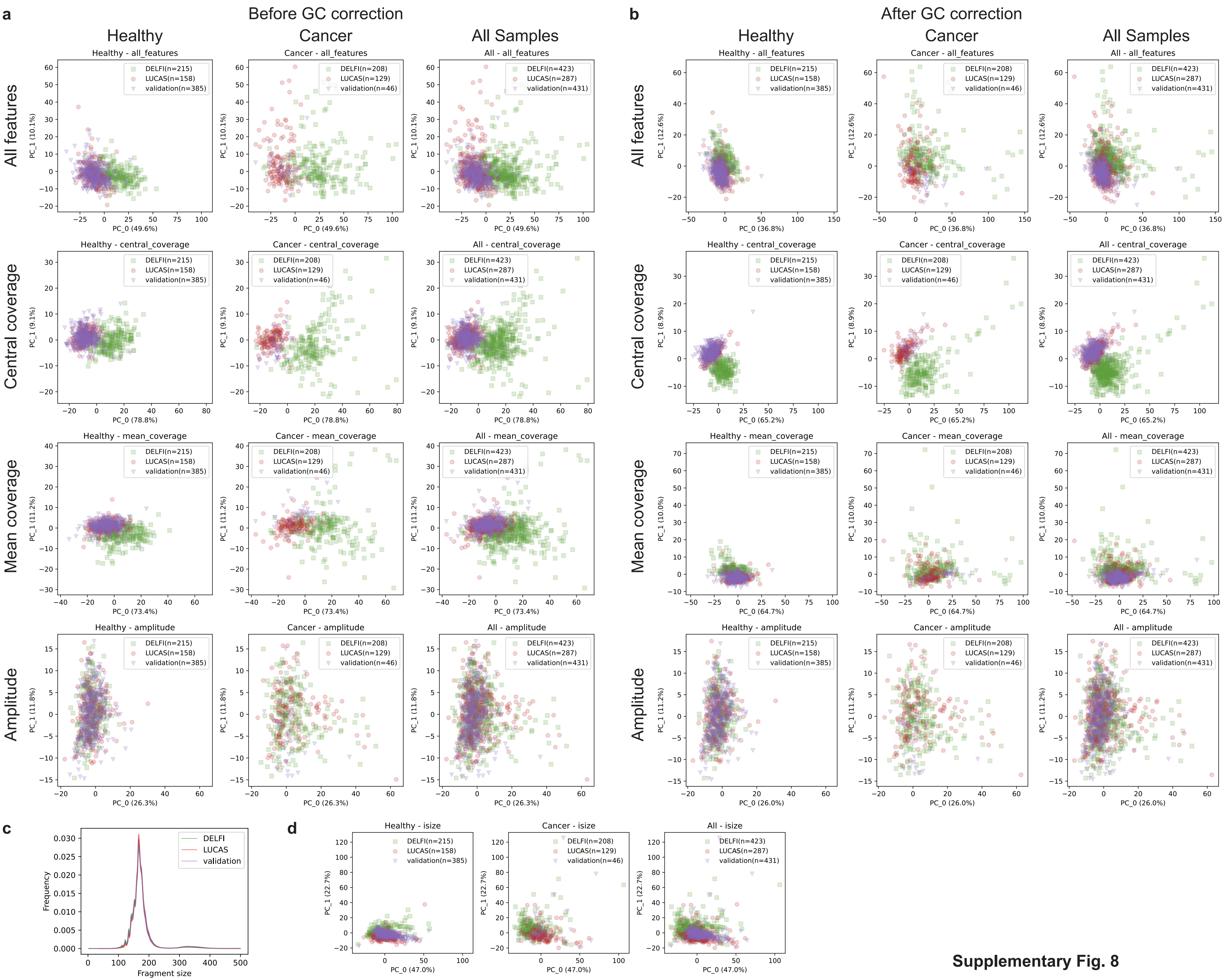

Supplementary Fig. 8

Supplementary Fig. 8: Principal component analysis (PCA) on Griffin features for cancer detection cohorts before GC correction **(a)** and after GC correction **(b)**. For each cancer detection cohort (DELFI, LUCAS, and LUCAS validation) Griffin analysis was performed on 30,000 TFBSs each for 270 TFs and 3 features (central coverage, mean coverage, and amplitude) were extracted from each profile for a total of 810 features. Top row, a PCA was performed on all features for all samples and the top two components were plotted for healthy samples from all three cohorts (left), cancer samples (middle) and all samples (right). The DELFI cohort clustered away from the other cohorts indicating systematic difference between the DELFI cohort and other cohorts. Next, PCA was performed separately on each of the 3 feature types: central coverage (second row), mean coverage (third row), and amplitude (bottom row) which revealed that the difference between the DELFI cohort and other cohorts was primarily due to differences in the central coverage. Percentage of variance explained by each PC is labeled on the axes. **(c)** Mean normalized fragment size profiles for the three cohorts. Shading indicates IQR. **(d)** PCA of the fragment size profiles in the three cohorts. Top two PCs are shown for healthy samples (left), cancer samples (middle), and all samples (right). Source data are provided as a Source Data file.

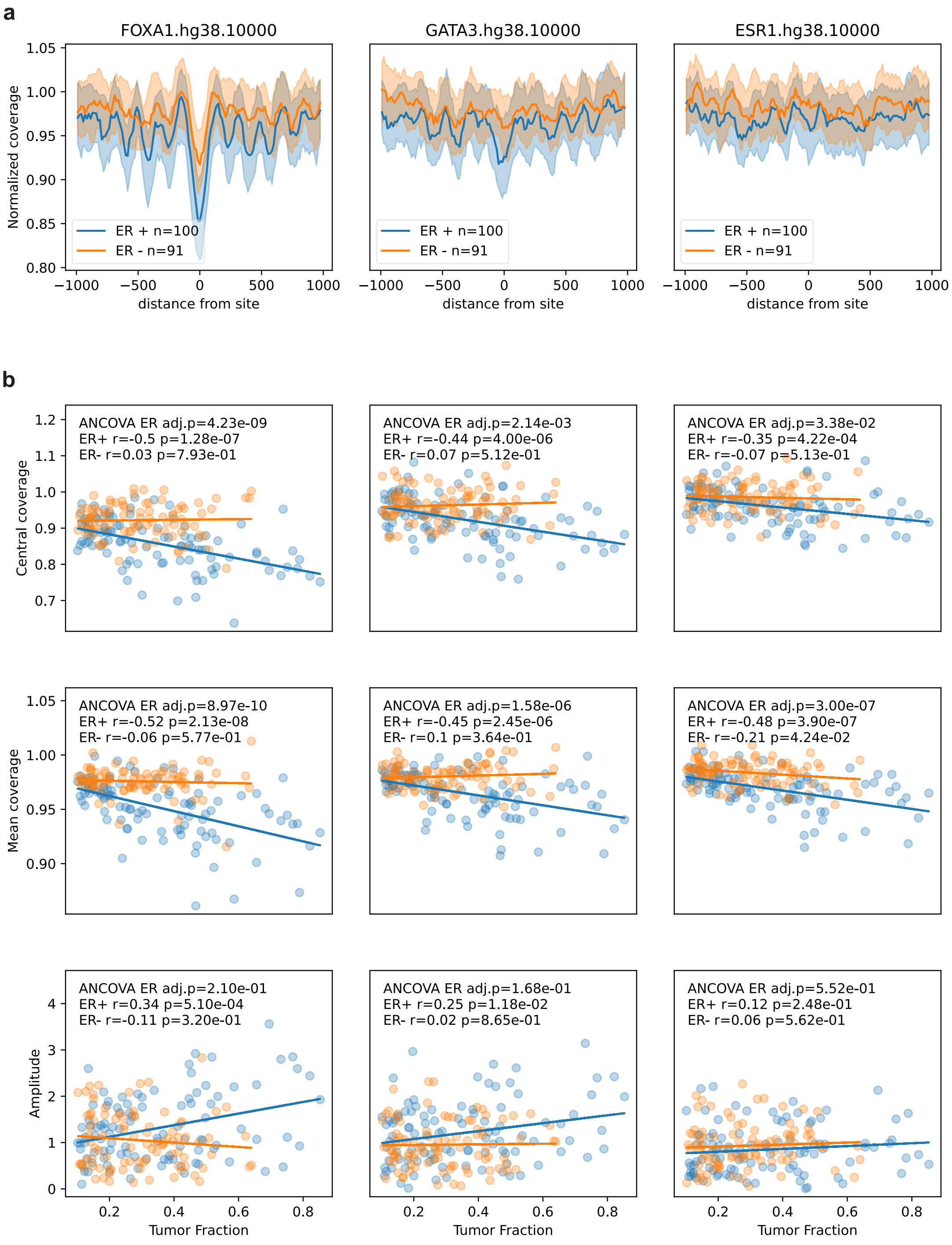

Supplementary Fig. 9

Supplementary Fig. 9: (a) Coverage profiles for the top 10,000 TFBSs for each of 3 key ER positive specific transcription factors, FOXA1, GATA3, and ESR1. Median ± IQR shown for 100 ER positive and 91 ER negative ULP-WGS MBC samples with ≥ 0.1 tumor fraction.[3] (**b)** Correlation between the three features and the tumor fractions for the coverage profiles shown in (a). Top row: central coverage, middle row: mean coverage, bottom row: amplitude. ANCOVA p-values (two sided) are shown for the ER status after accounting for the tumor fraction as a covariate. Benjamini-Hochberg FDR correction was used for multi test correction (See Methods). Pearson r and p-values (two-sided) for the correlation between tumor fraction and feature are shown for ER+ and ER- separately. Source data are provided as a Source Data file.

a

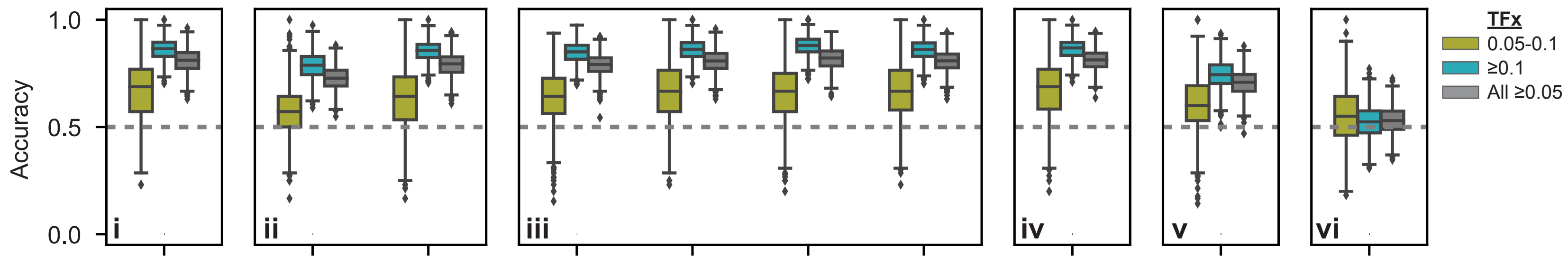

Fragment size	100-200bp	**35-150bp**	**35-500bp**	100-200bp	100-200bp	100-200bp	100-200bp	100-200bp	100-200bp	**All lengths**
GC correction	Yes	Yes	Yes	**No**	Yes	Yes	Yes	Yes	Yes	**No**
Mappability correction	No	No	No	No	**Yes**	No	No	No	No	No
CNA correction	No	No	No	No	No	**Yes**	No	No	No	**Yes**
GC correction strategy	Multi-length	Multi-length	Multi-length	**N/A**	Multi-length	Multi-length	**Single-length**	Multi-length	Multi-length	**N/A**
Amplitude included	Yes	Yes	Yes	Yes	Yes	Yes	Yes	**No**	Yes	Yes
Site Type	ATAC	ATAC	ATAC	ATAC	ATAC	ATAC	ATAC	ATAC	**TFBS**	**TFBS**
Tool used	Griffin	Griffin	Griffin	Griffin	Griffin	Griffin	Griffin	Griffin	Griffin	**Ulz et al.**

Accuracy (95% CI)

	finalized settings	short-fragment	all-fragment	no-GC-corr.	GC-map-corr.	CNA-corr.	single-len.-GC-corr.	no-amplitude	TFBS	Ulz et al.
**0.05-0.1 TFx**	0.69 (0.38-0.92)	0.57 (0.36-0.80)	0.64 (0.31-0.90)	0.64 (0.38-0.86)	0.67 (0.38-0.91)	0.67 (0.38-0.90)	0.67 (0.38-0.92)	0.69 (0.38-0.91)	0.60 (0.36-0.85)	0.55 (0.29-0.81)
**≥0.1 TFx**	0.86 (0.76-0.95)	0.79 (0.67-0.91)	0.86 (0.76-0.94)	0.85 (0.74-0.94)	0.86 (0.77-0.95)	0.88 (0.79-0.97)	0.86 (0.76-0.95)	0.87 (0.77-0.95)	0.74 (0.61-0.87)	0.52 (0.38-0.67)
**All ≥0.05 TFx**	0.81 (0.70-0.90)	0.73 (0.62-0.83)	0.79 (0.69-0.89)	0.79 (0.69-0.88)	0.81 (0.70-0.90)	0.82 (0.71-0.91)	0.81 (0.71-0.90)	0.81 (0.72-0.90)	0.71 (0.59-0.82)	0.53 (0.41-0.66)

b

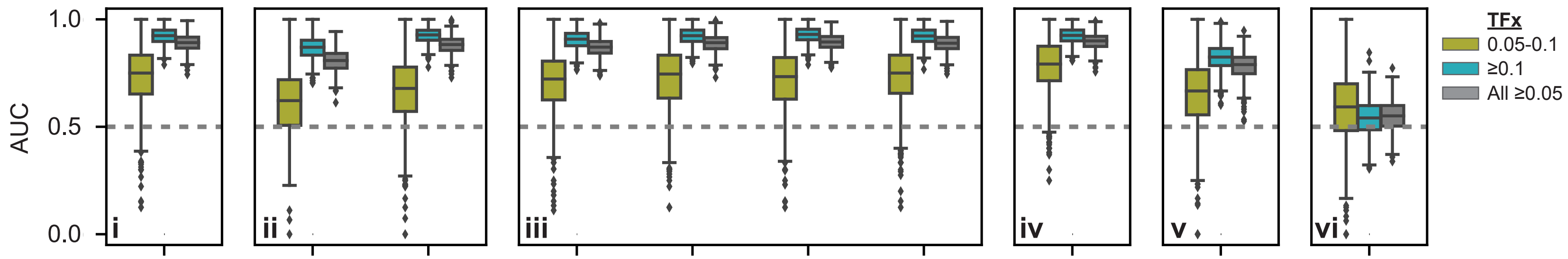

Fragment size	100-200bp	**35-150bp**	**35-500bp**	100-200bp	100-200bp	100-200bp	100-200bp	100-200bp	100-200bp	**All lengths**
GC correction	Yes	Yes	Yes	**No**	Yes	Yes	Yes	Yes	Yes	**No**
Mappability correction	No	No	No	No	**Yes**	No	No	No	No	No
CNA correction	No	No	No	No	No	**Yes**	No	No	No	**Yes**
GC correction strategy	Multi-length	Multi-length	Multi-length	**N/A**	Multi-length	Multi-length	**Single-length**	Multi-length	Multi-length	**N/A**
Amplitude included	Yes	Yes	Yes	Yes	Yes	Yes	Yes	**No**	Yes	Yes
Site Type	ATAC	ATAC	ATAC	ATAC	ATAC	ATAC	ATAC	ATAC	**TFBS**	**TFBS**
Tool used	Griffin	Griffin	Griffin	Griffin	Griffin	Griffin	Griffin	Griffin	Griffin	**Ulz et al.**

AUC (95% CI)

	finalized settings	short-fragment	all-fragment	no-GC-corr.	GC-map-corr.	CNA-corr.	single-len.-GC-corr.	no-amplitude	TFBS	Ulz et al.
**0.05-0.1 TFx**	0.75 (0.42-1.0)	0.62 (0.32-0.89)	0.68 (0.35-1.0)	0.72 (0.42-0.96)	0.75 (0.42-1.0)	0.73 (0.39-1.0)	0.75 (0.42-1.0)	0.79 (0.5-1.0)	0.67 (0.32-0.93)	0.59 (0.25-0.88)
**≥0.1 TFx**	0.92 (0.84-0.99)	0.87 (0.76-0.96)	0.93 (0.85-0.99)	0.91 (0.82-0.98)	0.92 (0.84-0.99)	0.93 (0.85-0.99)	0.92 (0.84-0.99)	0.93 (0.85-0.99)	0.82 (0.69-0.93)	0.54 (0.38-0.7)
**All ≥0.05 TFx**	0.89 (0.81-0.96)	0.81 (0.71-0.9)	0.88 (0.8-0.95)	0.87 (0.79-0.94)	0.89 (0.81-0.96)	0.89 (0.82-0.96)	0.89 (0.82-0.96)	0.9 (0.82-0.96)	0.79 (0.66-0.89)	0.55 (0.41-0.69)

Supplementary Fig. 10

Supplementary Fig. 10: Evaluation of various configurations and comparisons of Griffin for ER status prediction. **(a)** Boxplots of the accuracy values for 1,000 bootstrap iterations of the logistic regression classifier using various configurations and comparisons of Griffin on the MBC cohort (n=139, 0.1x WGS data). Accuracy is shown for patients grouped by tumor fraction (TFx), 0.05 – 0.1 (ER+, n=24; ER-, n=14) and ≥ 0.1 (ER+, n=50; ER-, n=51), and for all patients with ≥0.05 TFx. The boxed range represents the median ± IQR, whiskers represent the range of the non-outlier data (maximum extent is 1.5x the IQR). Outliers are shown as grey diamonds. **(i)** finalized Griffin configuration with parameters settings and median accuracy for the 1,000 bootstrap iterations (with 95% CI) listed underneath for comparison to other configurations. Logistic regression model was trained on the differential ATAC features (total of 12 features), see methods. **(ii)** same Griffin analysis as in (i) but using two different fragment size ranges, short fragments which are known to be enriched in cancer[7,8] (35-150bp) and a wider range of fragment sizes encompassing most cfDNA fragments (35-500bp). **(iii)** Same Griffin analysis as in (i) but without GC correction (left), with an added mappability correction step (middle left), with an added CNA correction step (middle right), and with a different GC correction approach using a single fragment length (right). **(iv)** Same as (i) but with the amplitude features excluded from the model (only used central coverage and mean coverage). **(v)** Griffin with standard parameters from (i) applied to the top 30,000 TFBSs for 270 TFs. Features were extracted and dimensionality was reduced with PCA using the same approach as described in the methods for cancer detection (see methods). **(vi)** Regression model on the outputs of the nucleosome profiling pipeline developed by Ulz and colleagues. This pipeline uses all fragment sizes (the vast majority of which are between 35 and 500bp) and collects coverage profiles around the top 1,000 sites for 504 transcription factors and extracts one feature (High frequency range) per feature. Dimensionality was reduced with PCA using the same approach as described in the methods for cancer detection using TFBSs and the top features were put into the logistic regression model. **(b)** Same as (a) but showing AUC rather than accuracy. Source data are provided as a Source Data file.

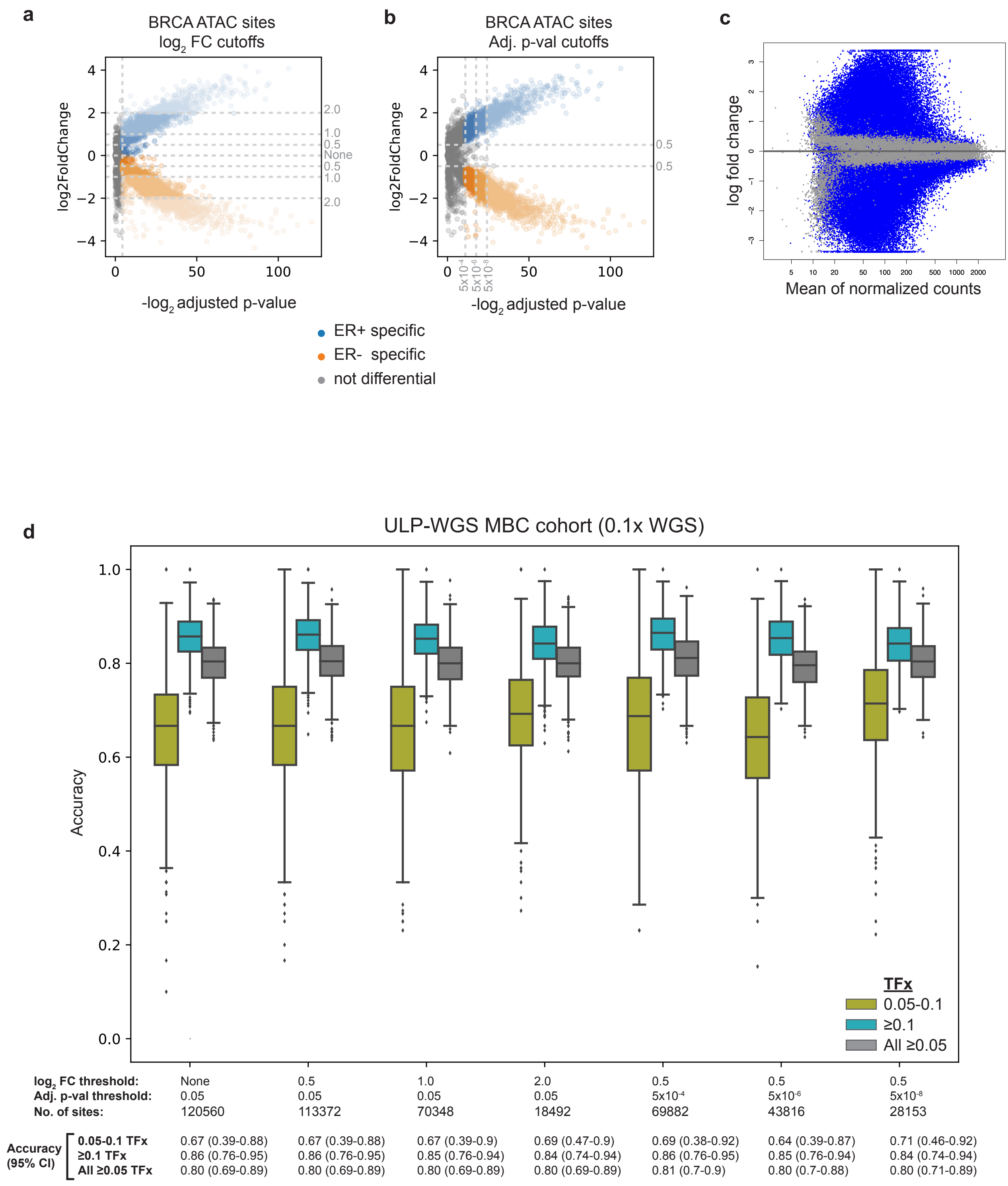

Supplementary Fig. 11

Supplementary Fig. 11: Evaluation of different cutoffs for differential ATAC site selection. **(a)** ER+ and ER- differential open chromatin sites were selected from assay for transposase-accessible chromatin using sequencing (ATAC-seq) data from ER+ (n=44) and ER- (n=15) breast cancer (BRCA) tumors in The Cancer Genome Atlas (TCGA).[9] Differential sites were identified using the DESeq2 software[10] which uses a Wald test with Benjamini-Hochberg FDR correction to calculate the adjusted p-value and $\log_2$ fold-change (FC) for each site. The butterfly plot displays the adjusted p-values and $\log_2$ fold-change values for ATAC sites. Several different cutoffs (None, 0.5, 1.0, 2.0) were considered and are shown with dashed lines. For all $\log_2$ FC cutoffs, an adjusted p-value cutoff of $5x10^{-2}$ was also used. Sites that met the criteria for being differential are shaded in blue (ER+) or orange (ER-). **(b)** Same as (a) but with various adjusted p-value cutoffs from DESeq2 ($5x10^{-4}$, $5x10^{-6}$,$5x10^{-8}$). For all cutoffs, a $\log_2$ fold-change cutoff of 0.5 was also used. **(c)** M-A plot showing the relationship between number of read counts at the sites and $\log_2$ fold change. Output from DESeq2. **(d)** Boxplot of accuracy values for 1,000 bootstrap iterations of the ER status prediction logistic regression model on the ULP-WGS MBC cohort using different DESeq2 cutoffs for selecting differential sites. Accuracy is shown for patients grouped by tumor fraction (TFx), 0.05 – 0.1 (ER+, n=24; ER-, n=14) and ≥ 0.1 (ER+, n=50; ER-, n=51), and for all patients with ≥0.05 TFx. 95% CIs were obtained by bootstrapping. The performance was generally similar for all cutoffs but there was a slightly higher performance when using a $\log_2$ FC of 0.5 and an adjusted p-value cutoff of $5x10^{-4}$ so this cutoff was used for further analysis. The boxed range represents the median ± IQR, whiskers represent the range of the non-outlier data (maximum extent is 1.5x the IQR). Outliers are plotted as grey diamonds. Source data are provided as a Source Data file.

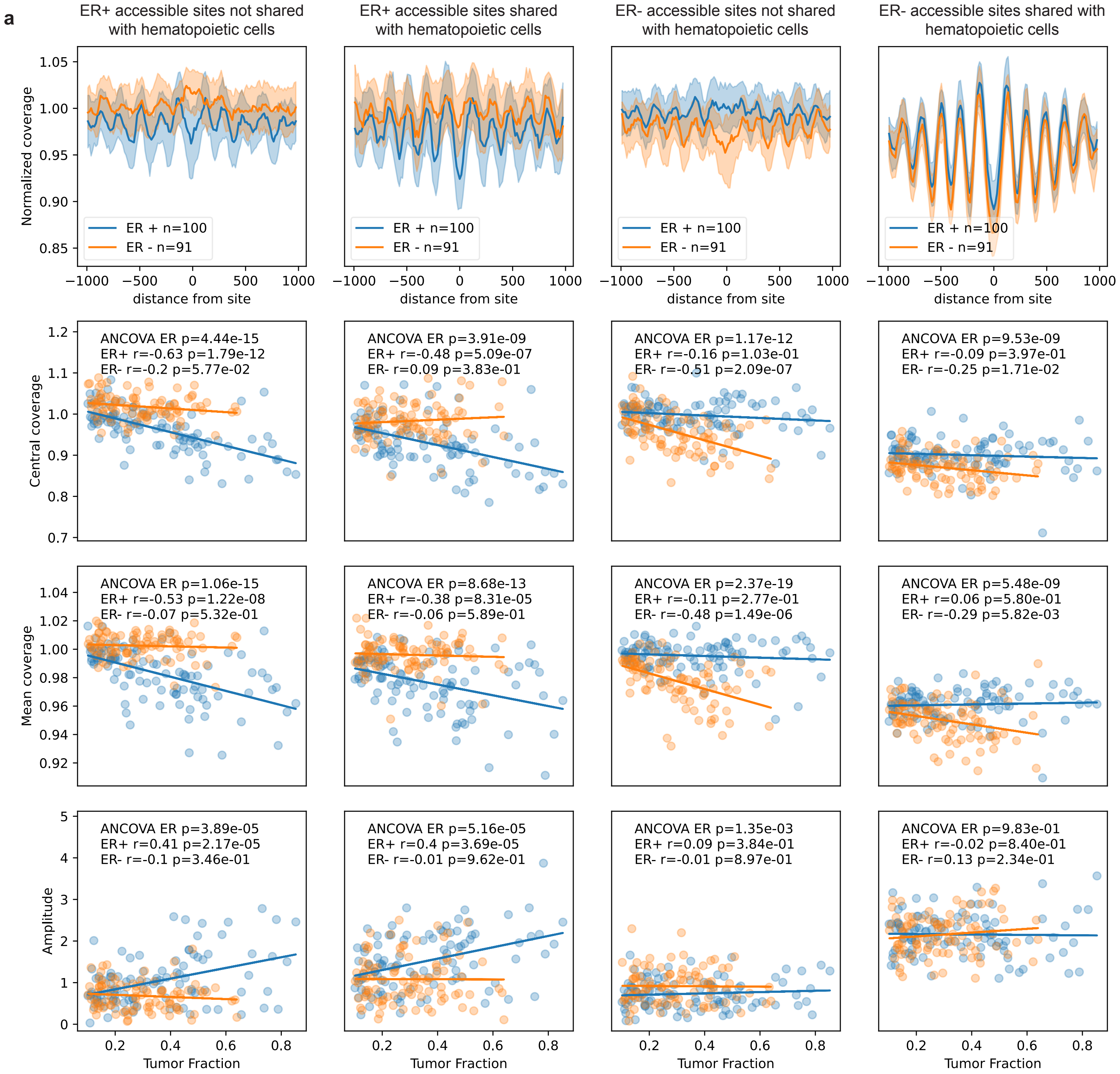

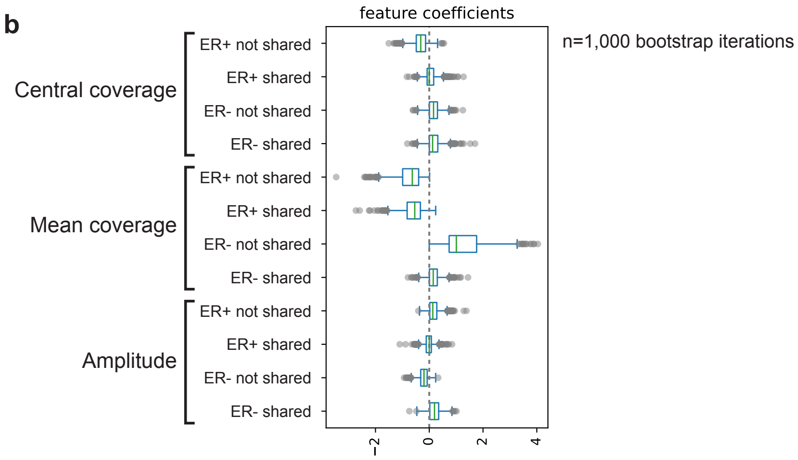

Supplementary Fig. 12

Supplementary Fig. 12: Differential ATAC seq features identified using the optimal $5x10^{-4}$ adjusted p-value cutoff from DESeq2 **(a)** First row: Griffin coverage profiles for ER subtype differential ATAC-seq sites in ER+ (n=100) and ER- (n=91) ULP-WGS MBC samples with ≥ 0.10 tumor fraction.[3] Median +/- IQR is shown. Second, third, and fourth rows: correlation between tumor fraction and central coverage (second row), mean coverage (third row), and amplitude (fourth row), respectively, for ER+ (n=100) and ER- (n=91) samples. ANCOVA p-values (two sided) for the ER status after accounting for the tumor fraction are shown on the plots (See methods, ANCOVA). Pearson r and p-values (two sided) for the correlation between tumor fraction and feature are shown for ER+ and ER- separately. **(b)** Boxplot of logistic regression feature coefficients for each of 1,000 bootstrap iterations. 'not shared' sites are not shared with hematopoietic cells, 'shared' are shared with hematopoietic cells. The boxed range represents the median ± IQR, whiskers represent the range of the non-outlier data (maximum extent is 1.5x the IQR). Outliers are plotted in grey. Source data are provided as a Source Data file.

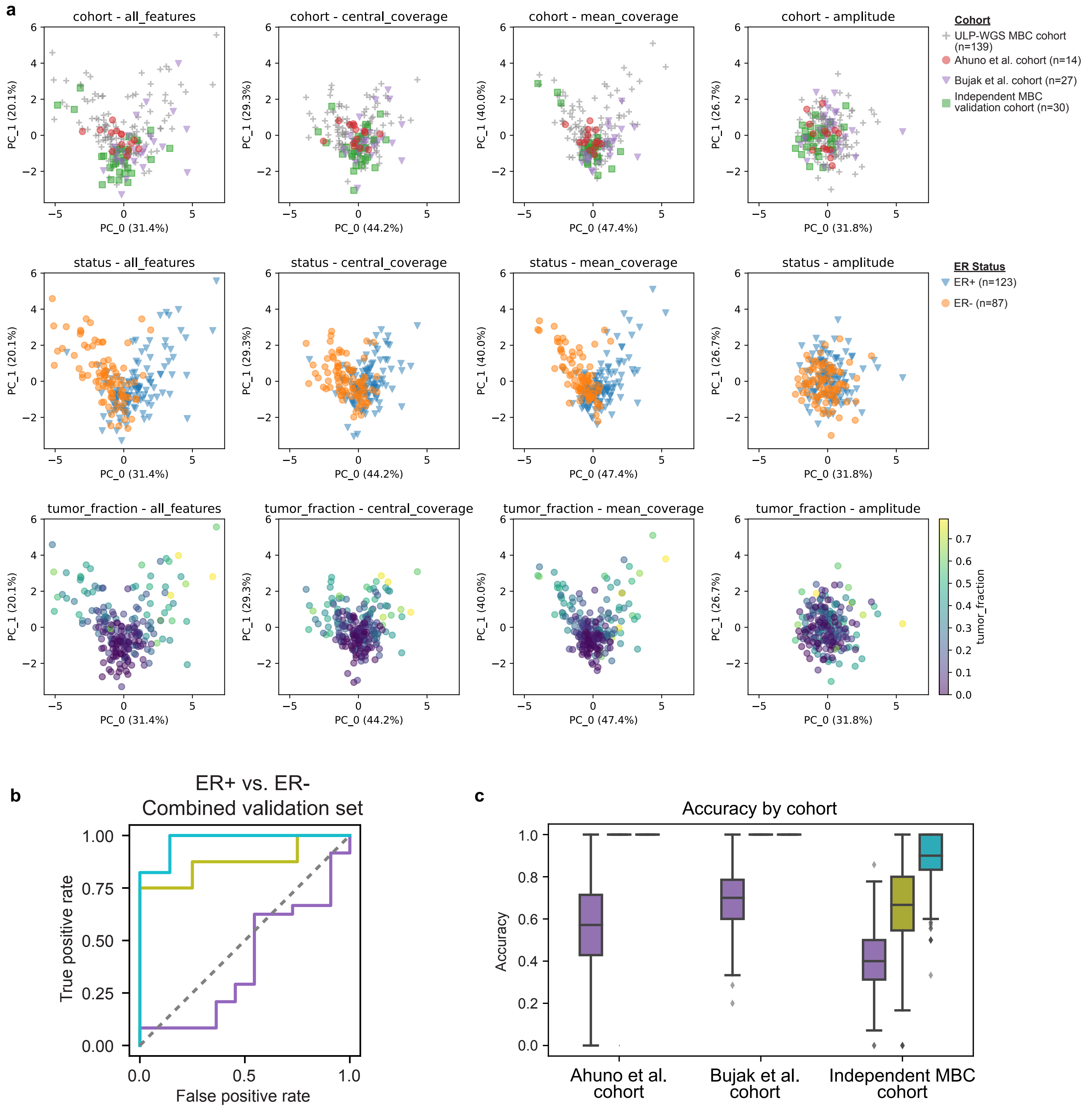

TFx	n	Accuracy	AUC
0-0.05	35	0.54 (0.38-0.70)	0.39 (0.19-0.61)
0.05-0.1	12	0.85 (0.60-1.00)	0.90 (0.60-1.00)
≥0.1	24	0.96 (0.86-1.00)	0.98 (0.89-1.00)

Ahuno et al. cohort

TFx	ER+	ER-	Accuracy
0-0.05	4	3	0.57 (0.14-1.00)
0.05-0.1	3	1	1.00 (1.00-1.00)
≥0.1	0	3	1.00 (1.00-1.00)

Bujak et al. cohort

TFx	ER+	ER-	Accuracy
0-0.05	13	0	0.70 (0.42-0.93)
0.05-0.1	2	0	1.00 (1.00-1.00)
≥0.1	12	0	1.00 (1.00-1.00)

Independent MBC Validation cohort

TFx	ER+	ER-	Accuracy
0-0.05	7	8	0.40 (0.15-0.67)
0.05-0.1	3	3	0.67 (0.25-1.00)
≥0.1	5	4	0.90 (0.67-1.00)

Supplementary Fig. 13

Supplementary Fig. 13: MBC validation set performance **(a)** Principal component analysis (PCA) on Griffin features for breast cancer samples from the initial ULP-WGS cohort and three validation cohorts. For each cohort Griffin analysis was performed on differential ATAC seq sites identified by DESeq2 using the $5x10^{-4}$ adjusted p-value cutoff. 3 features were extracted from each profile for a total of 12 features. A PCA was performed on all 12 features (first column), central coverage features only (second column), mean coverage features (third columns), or amplitude features (fourth column). This PCA was then colored by cohort (top row) to look for batch effects, but batch effects were not observed in the top two principal components. The PCA was also colored by ER status (second row), demonstrating that the first PC (PC_0, x axis) appears to correspond to status. Finally, the PCA was colored by tumor fraction (third row) indicating that the second PC (PC_1, y axis) corresponds to tumor fraction. Percentage of variance explained by each PC is labeled on the axes. **(b)** Receiver operator characteristic (ROC) curve for a logistic regression model predicting ER+ and ER- subtype on 71 breast cancer samples from the three validation cohorts[11,12]. Model was trained on the initial ULP-WGS cohort and applied to the three validation cohorts. ROC curve, accuracy and AUC are shown for all patients and for patients grouped by tumor fraction (TFx) similar to Figure 4e but including performance for samples below 0.05 TFx. 95% CIs were obtained by bootstrapping. For patients with multiple samples, the first sample was used. **(c)** Boxplot of the accuracy of the model on the validation cohorts grouped by tumor faction and cohort. Confidence intervals were obtained via bootstrapping (n=1000 bootstrap iterations). The boxed range represents the median ± IQR, whiskers represent the range of the non-outlier data (maximum extent is 1.5x the IQR). Outliers are plotted in grey. Source data are provided as a Source Data file.

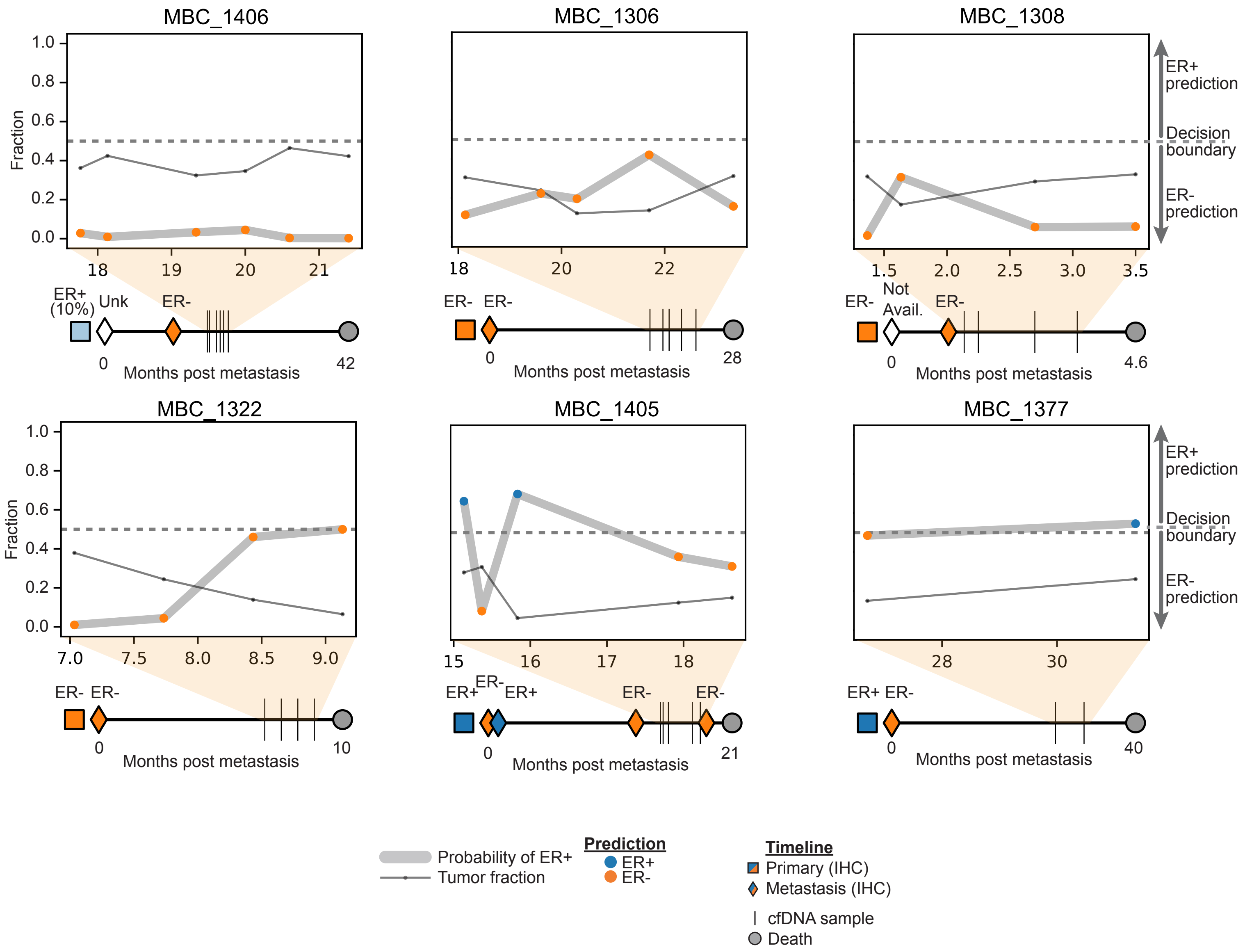

Supplementary Fig. 14

Supplementary Fig. 14: Timelines of disease progression for 6 patients with multiple metastatic biopsies and multiple cfDNA samples. ER+ prediction probability (thick grey line), and tumor fraction (thin grey line) are shown for all cfDNA samples that passed the >0.05 tumor fraction and 0.1x coverage thresholds. Decision boundary for ER+ (≥0.5) and ER- (<0.5) is indicated with dotted line. Timelines in months from metastatic diagnosis to death are shown for each patient. The square indicates primary status and timeline from primary is not to scale. Metastatic biopsies are shown with diamonds, blue indicates an ER+ biopsy, light blue an ER low biopsy (1-10% ER positive), orange an ER- biopsy, and white a biopsy where status was not available from clinical records. Source data are provided as a Source Data file.

References

1. Snyder, M. W., Kircher, M., Hill, A. J., Daza, R. M. & Shendure, J. Cell-free DNA Comprises an In Vivo Nucleosome Footprint that Informs Its Tissues-Of-Origin. *Cell* **164**, 57–68 (2016).
2. Dorritie, K. A., McCubrey, J. A. & Johnson, D. E. STAT transcription factors in hematopoiesis and leukemogenesis: opportunities for therapeutic intervention. *Leukemia* **28**, 248–257 (2014).
3. Adalsteinsson, V. A. *et al.* Scalable whole-exome sequencing of cell-free DNA reveals high concordance with metastatic tumors. *Nature Communications* **8**, 1324 (2017).
4. Liu, T. M., Lee, E. H., Lim, B. & Shyh-Chang, N. Concise Review: Balancing Stem Cell Self-Renewal and Differentiation with PLZF. *Stem Cells* **34**, 277–287 (2016).
5. Cristiano, S. *et al.* Genome-wide cell-free DNA fragmentation in patients with cancer. *Nature* **570**, 385–389 (2019).
6. Mathios, D. *et al.* Detection and characterization of lung cancer using cell-free DNA fragmentomes. *Nat Commun* **12**, 5060 (2021).
7. Mouliere, F. *et al.* Enhanced detection of circulating tumor DNA by fragment size analysis. *Science Translational Medicine* **10**, eaat4921 (2018).
8. Cristiano, S. *et al.* Genome-wide cell-free DNA fragmentation in patients with cancer. *Nature* **570**, 385–389 (2019).
9. Corces, M. R. *et al.* The chromatin accessibility landscape of primary human cancers. *Science* **362**, eaav1898 (2018).
10. Love, M. I., Huber, W. & Anders, S. Moderated estimation of fold change and dispersion for RNA-seq data with DESeq2. *Genome Biology* **15**, 550 (2014).

11. Ahuno, S. T. *et al.* Circulating tumor DNA is readily detectable among Ghanaian breast cancer patients supporting non-invasive cancer genomic studies in Africa. *NPJ Precis Oncol* **5**, 83 (2021).

12. Bujak, A. Z. *et al.* Circulating tumour DNA in metastatic breast cancer to guide clinical trial enrolment and precision oncology: A cohort study. *PLOS Medicine* **17**, e1003363 (2020).
