# Supplementary material for: A framework for clinical cancer subtyping from nucleosome profiling of cell-free DNA
Source: Nat Commun. 2022 Dec 3;13:7475. doi: 10.1038/s41467-022-35076-w (PMC9719521; doi:10.1038/s41467-022-35076-w)
Supplement: Supplementary file 3 — Description of Supplementary Data [file 41467_2022_35076_MOESM3_ESM.pdf]

Description of Additional Supplementary Files

Supplementary Data 1: S1_GC_content_around_TFBS
Summary of the GC content in the ±1,000bp window around the top 10,000 transcription factor binding sites (TFBSs) for 377 transcription factors (TFs). Related to Supplementary Fig. 1 b-d.

center: mean GC content in the window ±30bp from the TFBSs across the top 10,000 TFBSs
flanking: mean GC content in the window ±1000bp from the TFBSs (excluding the central ±30bp) across the top 10,000 TFBSs.
difference: center-flanking

Supplementary Data 2: S2_single_vs_multi_length_GC
Summary of the correlation between TFBS nucleosome profiling features and tumor fraction after GC correction with either a single fragment length model, or a multiple fragment length model in deep WGS (9-61x) from 14 castration resistant prostate cancer patients, two metastatic breast cancer patients, and two healthy donors. Two fragment size ranges were analyzed separately: short fragments (35-100bp) and nucleosome sized fragments (100-200bp). Related to Supplementary Fig. 2.

pearson_r_central_coverage.single_length.35-100bp Pearson r for the correlation between central coverage and tumor fraction for 35-100bp fragments using a single fragment length GC correction model.

pearson_pval_central_coverage.single_length.35-100bp p-value for the Pearson correlation between central coverage and tumor fraction for 35-100bp fragments using a single fragment length GC correction model.

lin_reg_RMSE_central_coverage.single_length.35-100bp The RMSE of the linear regression fit for the Pearson correlation between central coverage and tumor fraction for 35-100bp fragments using a single fragment length GC correction model.

These same three columns (pearson_r, pearson_pval, lin_reg_RMSE) are repeated for the other 2 features (mean_coverage and amplitude) and for the multi-fragment length correction strategy, and for nucleosome sized (100-200bp) fragments

Supplementary Data 3: S3_MBC_TFBS_vs_tumor_fraction
Correlations between TFBS features and tumor fraction in 191 MBC ULP-WGS samples.
For each of 377 TFs, the top 10,000 sites were analyzed using Griffin and the features were extracted from the composite profile. Related to Fig. 2f and Supplementary Figs. 1d, 3, and 4.

pearson-r_central_coverage.uncorrected: The Pearson correlation coefficient for the correlation between the central coverage feature and the tumor fraction before GC correction.
pearson-pval_central_coverage.uncorrected: The Pearson correlation p-value for the correlation between the central coverage feature and the tumor fraction before GC correction.
pearson-adjusted-p-val_central_coverage.uncorrected: p-value after Benjamini-Hochberg FDR correction.
central_cov_lin_reg_RMSE_uncorrected: The RMSE of the linear regression fit for the correlation between the central coverage feature and the tumor fraction before GC correction.

The remaining columns (F through AW) contain the same types of correlation values for the GC corrected analysis and for the other two features (mean coverage and amplitude). See Fig. 1b and Methods for further description of the features.

Supplementary Data 4: S4_differential_RNA_TFs
Differential expression analysis of TFs between blood and MBC. Related to Supplementary Fig. 4a

logFC_RNA_expr log2 fold-change of the RNA expression difference between blood and MBC. Positive has higher expression in blood, negative has higher expression in MBC. Absolute value > 1.5 required for a gene to be considered differentially expressed.

adj.P.Val_RNA_expr p-value after FDR correction of the RNA differential expression analysis.

group whether the gene was upregulated in blood, MBC, or not differentially expressed (pval > 0.05 or abs(log2FC) <1.5)

mean_overlaps_with_other type mean number of overlaps between the top 10,000 TFBSs for each differentially expressed transcription factor and the TBFS for TFs that are differentially upregulated in the opposite tissue type. (i.e. for each TF that was upregulated in blood, we looked at how many sites it shared with each of the 107 TFs that were upregulated in MBC and took the mean of these 107 values)

Supplementary Data 5: S5_HD_MAD_scores
Mean absolute deviation (MAD) of the TFBS features in 215 healthy donors.
For each of 377 TFs, the top 10,000 sites were analyzed with Griffin and the features were extracted from the composite profile. This table contains the MAD for each of three features: central coverage (central_cov), mean coverage (mean_cov) and amplitude, before (uncorrected) and after GC correction. Related to Fig. 2g and Supplementary Fig. 3c.

Supplementary Data 6: S6_DELFI_cohort_predictions
Cancer detection predictions and sample metadata for 215 healthy donors and 208 cancer patient samples in the DELFI cohort. Related to Fig. 3a.

sample: the sample type and patient ID.
original_bam_file: the name of the bam file downloaded from EGA.
sample_type: cancer type or healthy donor.
tumor_fraction: tumor fraction predicted by ichorCNA.
stage: cancer stage (I, II, III, IV) or blank for healthy donor; X = stage unknown; 0 = stage unknown.
median_probability_cancer_1-2x_WGS: the median probability of cancer across all bootstrap iterations for the original 1-2x WGS data.
cancer_predicted_1-2x_WGS: whether cancer was predicted in the median of all bootstrap iterations for the original 1-2x WGS data; 1= cancer predicted; 0=no cancer predicted. The mean(± CI) AUCs reported in the paper were derived from the AUCs of each bootstrap iteration, so they will not exactly match this median data.
median_probability_cancer_ULP_WGS: the median probability of cancer across all bootstrap iterations for the downsampled 0.1x WGS data.

cancer_predicted_ULP_WGS: whether cancer was predicted in the median of all bootstrap iterations for the downsampled 0.1x WGS data; 1= cancer predicted; 0=no cancer predicted. The median (± CI) AUCs reported in the paper were derived from the AUCs of each bootstrap iteration, so they will not exactly match this median data.

Supplementary Data 7: S7_DELFI_vs_LUCAS_ks-test
Results of Kolmogorov–Smirnov test (KS test) between feature values for healthy patients in the DELFI (n = 215) and LUCAS (n = 158) cohort. Healthy donors should have similar feature distributions, however, the large number of significant KS tests for the central_coverage and mean_coverage features indicate that there is a batch effect between these two cohorts.

central_coverage_pval: p-value from the KS test between the distribution of healthy donor central coverage values in the DELFI and LUCAS dataset for the top 30,000 TFBS for each of 270 TFs.
central_coverage_padj: adjusted KS test p-value after Benjamini-Hochberg correction.

These same 2 columns are repeated for the other two feature types: mean_coverage and amplitude

Supplementary Data 8: S8_LUCAS_cohort_predictions
Cancer detection predictions and sample metadata for 158 healthy donors and 129 cancer patient samples in the LUCAS cohort. Related to Fig. 3c.

sample: the sample ID
cancer_present: Whether or not the patient had cancer. 0 = no cancer; 1 = cancer.
original_bam_file: the name of the bam file downloaded from EGA.
tumor_fraction: tumor fraction predicted by ichorCNA.
stage: cancer stage (I, II, III, IV) or blank for healthy donor
median_probability_cancer_1-2x_WGS: the median probability of cancer across all bootstrap iterations for the original 1-2x WGS data.
cancer_predicted_1-2x_WGS: whether cancer was predicted in the median of all bootstrap iterations for the original 1-2x WGS data; 1= cancer predicted; 0=no cancer predicted. The mean(± CI) AUCs reported in the paper were derived from the AUCs of each bootstrap iteration, so they will not exactly match this median data.
median_probability_cancer_ULP_WGS: the median probability of cancer across all bootstrap iterations for the downsampled 0.1x WGS data.
cancer_predicted_ULP_WGS: whether cancer was predicted in the median of all bootstrap iterations for the downsampled 0.1x WGS data; 1= cancer predicted; 0=no cancer predicted. The median (± CI) AUCs reported in the paper were derived from the AUCs of each bootstrap iteration, so they will not exactly match this median data.

Supplementary Data 9: S9_LUCAS_Validation_cohort_pred
Cancer detection predictions and sample metadata for 385 healthy donors and 46 cancer patient samples in the LUCAS Validation cohort. Related to Fig. 3d.

sample: the sample ID
cancer_present: Whether or not the patient had cancer. 0 = no cancer; 1 = cancer.

original_bam_file: the name of the bam file downloaded from EGA.
tumor_fraction: tumor fraction predicted by ichorCNA.
stage: cancer stage (I, II, III-IV) or blank for healthy donor. Stages III and IV were combined due to the low number of samples in these two catergories.
probability_cancer_1-2x_WGS: the probability of cancer predicted by the logistic regression model trained on the LUCAS dataset for the original 1-2x WGS data.
cancer_predicted_1-2x_WGS: whether cancer was predicted for the original 1-2x WGS data; 1= cancer predicted; 0=no cancer predicted. The mean(± CI) AUCs reported in the paper were derived from the AUCs of each bootstrap iteration, so they will not exactly match this median data.
probability_cancer_ULP_WGS: the probability of cancer predicted by the logistic regression model trained on the LUCAS dataset for the downsampled 0.1x WGS data.
cancer_predicted_ULP_WGS: whether cancer was predicted for the downsampled 0.1x WGS data; 1= cancer predicted; 0=no cancer predicted. The median (± CI) AUCs reported in the paper were derived from the AUCs of each bootstrap iteration, so they will not exactly match this median data.

Supplementary Data 10: S10_ER_differential_TFBS_pvals
Results of the ANCOVA analysis on the nucleosome profiles around the top 10,000 TFBSs for 377 TFs in the MBC ULP cohort. Nucleosome profile feature (central coverage, mean coverage, or amplitude) was the dependent variable, primary tumor status ('status' in this supplementary table) was the independent variable ('between'), and tumor fraction was a covariate. P-values are shown before (pval) and after (adjusted-p-val) Benjamini-Hochberg FDR correction for both the 'status' variable and the 'tfx' variable. log2FC for the feature between ER+ and ER- is also shown to indicate which group has more accessibility (negative values indicate more accessibility in ER+ for central_coverage and mean_coverage and positive values indicate more accessibility for ER+ for amplitude). Related to Supplementary Fig. 9.

Supplementary Data 11: S11_ER_diff_ATAC_ thresholds
Number of ER subtype differentially accessible open chromatin sites based on various DESeq2 adjusted p-value and log2-fold-change cutoffs. Numbers of sites are categorized by those that had increased accessibility in each subtype (ER_positive and ER_negative) and further subdivided into those that did not overlap accessible sites in hematopoietic cells (specific) and those that overlapped accessible sites in hematopoietic cells (overlaps). Related to Supplementary Fig. 11.

Supplementary Data 12: S12_ER_differential_sites
The differential ATAC-seq sites used for ER status prediction. Related to Fig. 4a.

Peak_Name: The peak name used in the TCGA ATAC-seq dataset
pval: pvalue from DESeq2
padj: Adjusted p value from DESeq2
log2FoldChange: Adjusted log2 fold change from DESeq2
site_type: Which differential group this site belonged to (ER+ or ER- specific and shared or not shared with hematopoietic cells)
site_type: Which differential group this site belonged to (increased accessibility in ER+ or ER- and shared or not shared with hematopoietic cells)

<u>Supplementary Data 13</u>: S13_ATAC_TFBS_overlaps
Overlaps between ER differential ATAC-seq sites and TFBS.
Number of differential ATAC-seq binding sites in each group that overlapped the top 10,000 TFBS for each transcription factor.

TF_name: The name of the transcription factor
ER_positive_non_hematopoietic_18240_sites: Number of sites with increased accessibility in ER+ ATAC-seq that were not shared with hematopoietic cells (n=18,240) and overlapped with the top 10,000 TFBS for each of the 377 TFs. For example, of the 18,240 total sites that were specific to ER+ and not shared with hematopoietic cells, 1722 (9.4%) overlapped at least one of the top 10,000 TFBS for AHR.
ER_positive_hematopoietic_9930_sites: Overlaps between sites with increased accessibility in ER+ ATAC-seq that are shared with hematopoietic cells (n=9,930) and TFBS.
ER_negative_non_hematopoietic_19347_sites: Overlaps between sites with increased accessibility in ER- ATAC-seq that are not shared with hematopoietic cells (n=19,347) and TFBS.
ER_negative_hematopoietic_22365_sites: Overlaps between sites with increased accessibility in ER- ATAC seq that are shared with hematopoietic cells (n=22,365) and TFBS.

<u>Supplementary Data 14</u>: S14_MBC_subtyping
ER status prediction results for the MBC cohort. Metadata was abstracted from medical records. Related to Fig. 4c, d, f, g.

sample: Randomized breast cancer sample IDs.
patient_id: randomized breast cancer patient ID.
tumor_fraction: tumor fraction predicted by ichorCNA.
ulp_wgs_coverage: were obtained directly from the study by Adalsteinsson et al
primary_ER: the ER status of the primary tumor determined by IHC; + = ER positive; - = ER negative; low = low ER expression (1-10%, considered positive for the classifier); none=status not known/IHC not performed.
met_ER: the ER status of the metastasis determined by IHC. If multiple met statuses were known, the last before cfDNA was used.
ER_switch: whether the ER status changed between the primary and metastasis; same = status did not change; loss = ER+/low primary and ER- metastasis; gain = ER- primary and ER+/low metastasis; unknown = either the primary or metastasis status is not known.
ER_status: the status used as the ground truth when training and testing the classifier; the primary status was used if the metastatic status was not known.
binary_ER_status: the ER status used for binary classification; ER low was labeled positive.
first_sample: denotes the earliest sample (or only sample) from each patient within this group of 254 samples (patient may have other samples that did not pass tumor fraction or coverage thresholds and were not used for this study).
median_probability_ER+: the predicted probability that a sample is ER+; this is the median probability value from all bootstraps where a given sample was in the test set. The mean(± CI) AUCs and accuracies reported in the paper were derived from the AUCs and accuracies of each bootstrap iteration, so they will not exactly match this median data.
Median_prediction: the median prediction from all bootstraps where a given sample was in the test set; (1 = ER+; 0 = ER-).

<u>Supplementary Data 15</u>: S15_MBC_validation_subtyping

ER status prediction results for the three validation cohorts. Related to Fig. 4e and Supplementary Fig. 13.

Dataset: Indicates the cohorts – Independent MBC cohort, Bujak et al. cohort, or Ahuno et al. cohort.
Tumor_fraction: Estimated by ichorCNA
Coverage: Genome wide depth of coverage of the ULP-WGS or downsampled data. Calculated using picard WGS metrics.
Sample_order: Order that blood samples were drawn, first sample was used for calculating performance.
HR status: Breast cancer hormone status – triple negative breast cancer (TNBC) or hormone positive (HR+) and HER2-negative (HER-). Provided for the Independent MBC cohort, all HR+ were confirmed to also be ER+.
ER status: Estrogen receptor status (ER+ or ER-). Provided for the Bujak et al. and Ahuno et al. cohorts.
Status: Label used for assessing the performance of the classifier (1= ER+; 0 = ER-)
Probability_ER: The predicted probability that a sample is ER+ using the model trained on the MBC ULP cohort and applied to these validatin cohorts. The mean(± CI) AUCs and accuracies reported in the paper were derived from the AUCs and accuracies of each bootstrap iteration, so they will not exactly match this median data.
Prediction: Classification predicted by trained model; (1 = ER+; 0 = ER-).

<u>Supplementary Data 16</u>: S16_MBC_timelines
Metastatic breast cancer (MBC) cohort tumor biopsy clinical annotations and ctDNA results for 14 patients with longitudinal plasma collected. Related to Fig. 4g.

Days_from_primary, days_from_met, months_from_met: Time in days or months that the sample (primary tumor, metastatic tumor, plasma) was obtained from initial primary or initial metastatic diagnosis (day 0).
Tumor_fraction: ichorCNA value estimated in the original study by Adalsteinsson et al.
Patient_ER_status_label: ER status determined by IHC for the most recent tumor biopsy closest in time to the first available plasma sample (denoted by 1 in column: last_before_cfDNA).
Median_probability_ER+: The predicted probability that a cfDNA sample is ER+ as determined by Griffin; this is the median probability value from all bootstraps where a given sample was in the test set. The mean(± CI) AUCs and accuracies reported in the paper were derived from the AUCs and accuracies of each bootstrap iteration, so they will not exactly match this median data.
Location: Anatomical location of the biopsy abstracted from clinical annotations of the tumor histopathology
ER_status_biopsy: The ER status of a given biopsy. "+" denotes ER+; "-" denotes ER-; "low" denotes ER-low. This is a simplified version of the information in column 'ER_status_notes'.
last_before_cfDNA: Denotes whether a biopsy was the last tumor biopsy prior to the first plasma sample used for classification. (1 = last tumor biopsy; 0 = not the last tumor biopsy)
ER_status_notes: Abstracted clinical annotation from the tumor histopathology.
DeathCensor: Denotes whether the patient died during the follow up window; 1 = patient died, 0 = patient was alive at the time of the last follow up.
