# Supplementary material for: A framework for clinical cancer subtyping from nucleosome profiling of cell-free DNA
Source: Nat Commun. 2022 Dec 3;13:7475. doi: 10.1038/s41467-022-35076-w (PMC9719521; doi:10.1038/s41467-022-35076-w)
Supplement: Supplementary file 20 — Reporting Summary [file 41467_2022_35076_MOESM20_ESM.pdf]

Corresponding author(s): Gavin Ha
Last updated by author(s): Aug 19, 2022

Reporting Summary

Nature Portfolio wishes to improve the reproducibility of the work that we publish. This form provides structure for consistency and transparency in reporting. For further information on Nature Portfolio policies, see our Editorial Policies and the Editorial Policy Checklist.

Statistics

For all statistical analyses, confirm that the following items are present in the figure legend, table legend, main text, or Methods section.

n/a	Confirmed	
☐	☒	The exact sample size (*n*) for each experimental group/condition, given as a discrete number and unit of measurement
☐	☒	A statement on whether measurements were taken from distinct samples or whether the same sample was measured repeatedly
☐	☒	The statistical test(s) used AND whether they are one- or two-sided<br>*Only common tests should be described solely by name; describe more complex techniques in the Methods section.*
☐	☒	A description of all covariates tested
☐	☒	A description of any assumptions or corrections, such as tests of normality and adjustment for multiple comparisons
☐	☒	A full description of the statistical parameters including central tendency (e.g. means) or other basic estimates (e.g. regression coefficient) AND variation (e.g. standard deviation) or associated estimates of uncertainty (e.g. confidence intervals)
☐	☒	For null hypothesis testing, the test statistic (e.g. *F*, *t*, *r*) with confidence intervals, effect sizes, degrees of freedom and *P* value noted<br>*Give P values as exact values whenever suitable.*
☒	☐	For Bayesian analysis, information on the choice of priors and Markov chain Monte Carlo settings
☒	☐	For hierarchical and complex designs, identification of the appropriate level for tests and full reporting of outcomes
☐	☒	Estimates of effect sizes (e.g. Cohen's *d*, Pearson's *r*), indicating how they were calculated

Our web collection on statistics for biologists contains articles on many of the points above.

Software and code

Policy information about availability of computer code

Data collection

No software was used for data collection.

Data analysis

Picard (2.18.29) was used for unmapping reads prior to realignment, marking duplicate reads, and calculating sequencing metrics
BWA-MEM (0.7.17) was used for realignments
GATK (4.1.0.0) was used for base recalibration
Samtools (1.9) was used for sorting bam files
ichorCNA (commit version 15b1d336) was used for tumor fraction estimation (downloaded from https://github.com/GavinHaLab/ichorCNA on 11-20-2020)
Griffin (0.2.0) pipeline is available on Github (https://github.com/adoebley/Griffin/)
Analyses were performed using Python 3.7.4. Analysis scripts are available on github (https://github.com/adoebley/Griffin_analyses)

python packages used for Griffin:
pysam 0.15.4
pyBigWig 0.3.17
pandas 1.3.2
numpy 1.21.2
scipy 1.7.1
matplotlib 3.4.1
snakemake 5.5.4

Other analysis packages used:
pandas 1.3.2
pysam 0.15.4

numpy 1.21.2
scipy 1.7.1
pyyaml 5.3.1
pyBigWig 0.3.17
pybedtools 0.8.0
matplotlib 3.4.1
scikit-learn 0.23.2
pingouin 0.5.1
pybedtools 0.8.0
statsmodels 0.13.2

For manuscripts utilizing custom algorithms or software that are central to the research but not yet described in published literature, software must be made available to editors and reviewers. We strongly encourage code deposition in a community repository (e.g. GitHub). See the Nature Portfolio guidelines for submitting code & software for further information.

Data

Policy information about availability of data

All manuscripts must include a data availability statement. This statement should provide the following information, where applicable:

- Accession codes, unique identifiers, or web links for publicly available datasets
- A description of any restrictions on data availability
- For clinical datasets or third party data, please ensure that the statement adheres to our policy

New WGS of cfDNA datasets in this study:
The Independent MBC Cohort data generated in this study have been deposited in dbGaP under accession code xxx (in process of obtaining).

Published data from previous studies
ATAC-seq used in this study is publicly available and can be downloaded from: https://atacseq.xenahubs.net/ (See methods for more detail)
DHS (DNAse hypersensitivity sites) used in this study is publicly available and can be downloaded from: https://zenodo.org/record/3838751/files/DHS_Index_and_Vocabulary_hg38_WM20190703.txt.gz
GTRD (ChIP seq): version 19.10, downloaded from https://gtrd.biouml.org/downloads/19.10/chip-seq/Homo%20sapiens_meta_clusters.interval.gz
Xena (RNA seq): version 2016-04-12, accessed at https://toil-xena-hub.s3.us-east-1.amazonaws.com/download/TcgaTargetGtex_RSEM_Hugo_norm_count.gz (analyzed with UCSC Xena, see methods for details)

Published WGS of cfDNA from previous studies:
dbGaP accession phs001417.v1.p1 [https://www.ncbi.nlm.nih.gov/projects/gap/cgi-bin/study.cgi?study_id=phs001417.v1.p1]
dbGaP accession phs002387.v1.p1 [https://www.ncbi.nlm.nih.gov/projects/gap/cgi-bin/study.cgi?study_id=phs002387.v1.p1])
EGA dataset ID EGAD00001005339 [https://ega-archive.org/datasets/EGAD00001005339]
EGA dataset ID EGAD00001007796 [https://ega-archive.org/datasets/EGAD00001007796]
NCBI BioProject accession number PRJNA578569 [https://www.ncbi.nlm.nih.gov/bioproject/PRJNA578569]

Field-specific reporting

Please select the one below that is the best fit for your research. If you are not sure, read the appropriate sections before making your selection.

☒ Life sciences ☐ Behavioural & social sciences ☐ Ecological, evolutionary & environmental sciences

For a reference copy of the document with all sections, see nature.com/documents/nr-reporting-summary-flat.pdf

Life sciences study design

All studies must disclose on these points even when the disclosure is negative.

Sample size

For cancer detection analysis, three datasets were used: DELFI (208 cancer and 215 healthy samples), LUCAS cohort (129 cancer and 158 healthy), LUCAS Validation cohort (46 cancer and 385 healthy samples). Training was performed on DELFI and LUCAS cohorts, which both have sufficient numbers of both classes (cancer vs healthy) to train a regularized logistic regression model with cross-validation performance of 0.76 AUC with conf interval 0.67-0.83. Independent testing of the trained models was performed on the LUCAS Validation cohort, which had enough cancer samples with performance of 0.86 AUC (CI 0.78-0.91).

For breast cancer subtype prediction, we used four datasets: MBC cohort (74 ER+, 65 ER-; 139 total), Validation cohorts (25 ER+, 11 ER-). For training, the MBC cohort provided sufficient numbers for both classes (ER+ vs ER-) as we performed 1000 iterations of randomized bootstrapping to generate datasets for cross-validation performance of 0.89 with confidence interval 0.81-0.96, which was acceptable. For the validation cohorts, the performance was 0.96 AUC (CI: 0.88-1.00), which was acceptable.

Data exclusions

MBC samples were excluded from analysis if they had insufficient coverage, insufficient tumor fraction, single end reads, or unknown ER status (see methods).

Replication

Independent validation for cancer detection was performed on the LUCAS Validation cohort, which had a performance of 0.86 AUC (CI 0.78-0.91), confirming the ability of this application.

Independent validation for breast cancer subtyping was performed on three validation cohorts (combined) with performance 0.96 AUC (CI:

	0.88-1.00), which confirmed the performance and utility of this application.
Randomization	We do not have experimental groups
Blinding	For training cohorts, we required the labels for cancer detection (cancer vs healthy) and for breast cancer subtyping (ER+ vs ER-). For independent validation, we used all available samples provided with the datasets obtained from other sources. When evaluating performance on validation cohorts, we applied the trained model which did not involve any samples from the validation cohorts and thus will not subject to overfitting. We required known labels for the validation cohorts to evaluate performance in the validation study.

Reporting for specific materials, systems and methods

We require information from authors about some types of materials, experimental systems and methods used in many studies. Here, indicate whether each material, system or method listed is relevant to your study. If you are not sure if a list item applies to your research, read the appropriate section before selecting a response.

Materials & experimental systems

n/a	Involved in the study
☒	☐ Antibodies
☒	☐ Eukaryotic cell lines
☒	☐ Palaeontology and archaeology
☒	☐ Animals and other organisms
☐	☒ Human research participants
☒	☐ Clinical data
☒	☐ Dual use research of concern

Methods

n/a	Involved in the study
☒	☐ ChIP-seq
☒	☐ Flow cytometry
☒	☐ MRI-based neuroimaging

Human research participants

Policy information about studies involving human research participants

Population characteristics	Data from human participants were obtained from prior studies through controlled access sequencing repositories/ databases. These patients were de-identified and population characteristics were not available.
Recruitment	No new participants were recruited for this study.
Ethics oversight	This study was approved by an IRB at the Dana Farber Cancer Research institute (IRB protocol identifiers 05-246, 09-204, 12-431) and Ohio State University (2007C0066, 2018C0211)

Note that full information on the approval of the study protocol must also be provided in the manuscript.
